# Inhibiting the alarmin‐driven hematopoiesis‐stromal cell crosstalk in primary myelofibrosis ameliorates bone marrow fibrosis

**DOI:** 10.1002/hem3.70179

**Published:** 2025-08-14

**Authors:** Hélène F. E. Gleitz, Stijn N. R. Fuchs, Inge A. M. Snoeren, Charlotte Boys, James Nagai, Hector Tejeda‐Mora, Vanessa Klöker, Jessica E. Pritchard, Iris J. Bakker, Marta Gargallo Garasa, Eric Bindels, Julio Saez‐Rodriguez, Thomas Vogl, Rafael Kramann, Aurélien Dugourd, Ivan G. Costa, Rebekka K. Schneider

**Affiliations:** ^1^ Department of Developmental Biology Erasmus Medical Center Rotterdam The Netherlands; ^2^ Oncode Institute Erasmus Medical Center Cancer Institute Rotterdam The Netherlands; ^3^ Department of Cell and Tumor Biology, Faculty of Medicine University Hospital RWTH Aachen Aachen Germany; ^4^ Institute for Computational Biomedicine, Faculty of Medicine, and Heidelberg University Hospital Heidelberg University Heidelberg Germany; ^5^ Institute for Computational Genomics, Faculty of Medicine RWTH Aachen University Aachen Germany; ^6^ Department of Hematology Erasmus Medical Center Cancer Institute Rotterdam The Netherlands; ^7^ Institute of Immunology University of Münster Münster Germany; ^8^ Institute of Experimental Medicine and Systems Biology, Medical Faculty RWTH Aachen University Aachen Germany; ^9^ Division of Nephrology and Clinical Immunology, Medical Faculty RWTH Aachen University Aachen Germany; ^10^ Department of Internal Medicine, Nephrology and Transplantation Erasmus Medical Center Rotterdam The Netherlands

## Abstract

Inflammation from the hematopoietic compartment is a critical driver of fibrosis and cytopenias in myeloproliferative neoplasms (MPNs). We previously demonstrated that tasquinimod ameliorates the MPN phenotype, reducing splenomegaly and normalizing fibrosis in a JAK2V617F‐driven preclinical model. Using bulk RNA sequencing, we now show that tasquinimod primarily targets the malignant JAK2V617F hematopoietic clone, particularly affecting megakaryocytes and monocytes. Tasquinimod downregulates pro‐proliferative pathways, MYC targets, and mTORC signaling, while increasing apoptosis in particularly in JAK2V617F mutant cells. Our data reveal that tasquinimod reverses TGFβ‐driven fibrotic reprogramming of megakaryocytes and monocytes. This reversal is crucial for mitigating the pro‐fibrotic interactions and signaling in the BM, thereby decreasing the activation of stromal cells. Coculture experiments confirm that direct interaction between JAK2V617F hematopoietic cells and mesenchymal stromal cells upregulates S100A8 in stromal cells, independent of TGFβ alone. In line, genetic ablation of S100A9 in the hematopoietic but not stromal compartment significantly improves the MPN phenotype and normalizes BM fibrosis. Our data highlight the hematopoietic origin of the inflammatory signals driving fibrosis. These insights pave the way for potential therapeutic strategies targeting inflammatory signaling pathways in MPN to mitigate fibrosis and improve patient outcomes.

## Graphical abstract

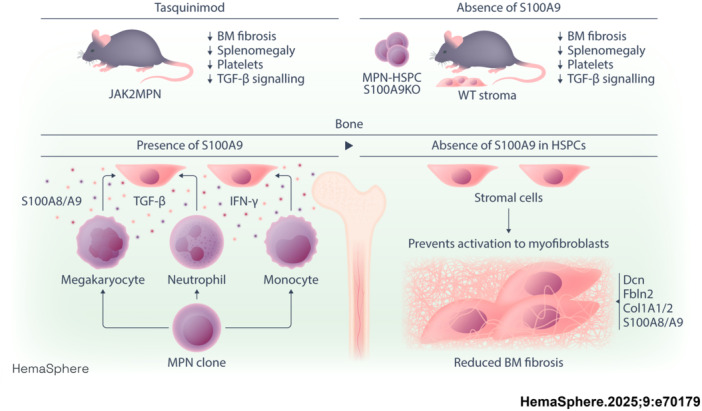

## INTRODUCTION

Primary myelofibrosis (PMF) is a myeloproliferative neoplasm (MPN) that arises from clonal proliferation of mutated hematopoietic stem cells (HSCs) and leads to progressive bone marrow (BM) fibrosis, resulting in extramedullary hematopoiesis (typically in the spleen), BM failure, and ultimately death. MPN driver mutations occur in HSCs in *JAK2*, *CALR*, and *MPL*, which are mutually exclusive, and JAK2^V617F^ is the most prevalent mutation in PMF. Previous work established roles for not only the hematopoietic compartment but also non‐hematopoietic stroma in the development and progression of PMF.^1^, ^2^, ^3^, ^4^, ^5^ We demonstrated using single‐cell RNAseq a disease‐specific upregulation of the alarmin complex S100A8/S100A9 in fibrosis‐driving mesenchymal stromal cell (MSC) populations.^1^ Importantly, targeting these alarmins with the small molecular oral inhibitor tasquinimod stopped the progression of fibrosis in murine JAK2^V617F^ PMF models. The questions remained whether the effect of alarmin inhibition of tasquinimod is more relevant in hematopoietic or stromal cells and how tasquinimod affects the hematopoiesis‐stromal cell crosstalk.

Here, we analyzed the effect of tasquinimod on hematopoietic and stromal cells using RNA sequencing in a JAK2V617F murine PMF model. We systematically analyzed the effect of stromal versus hematopoietic S100A9 inhibition using a genetic knockout model and determined the consequences of S100A9 overexpression using a genetic knock‐in model and lentiviral overexpression. Our findings shed light on the intricate interplay between hematopoietic and stromal cells in PMF and provide insights into the therapeutic potential of targeting alarmins in this disease context.

## RESULTS

### Tasquinimod affects TGFβ‐driven ECM remodeling in megakaryocytes and monocytes in JAK2^V617F^‐driven PMF

**Figure 1:**
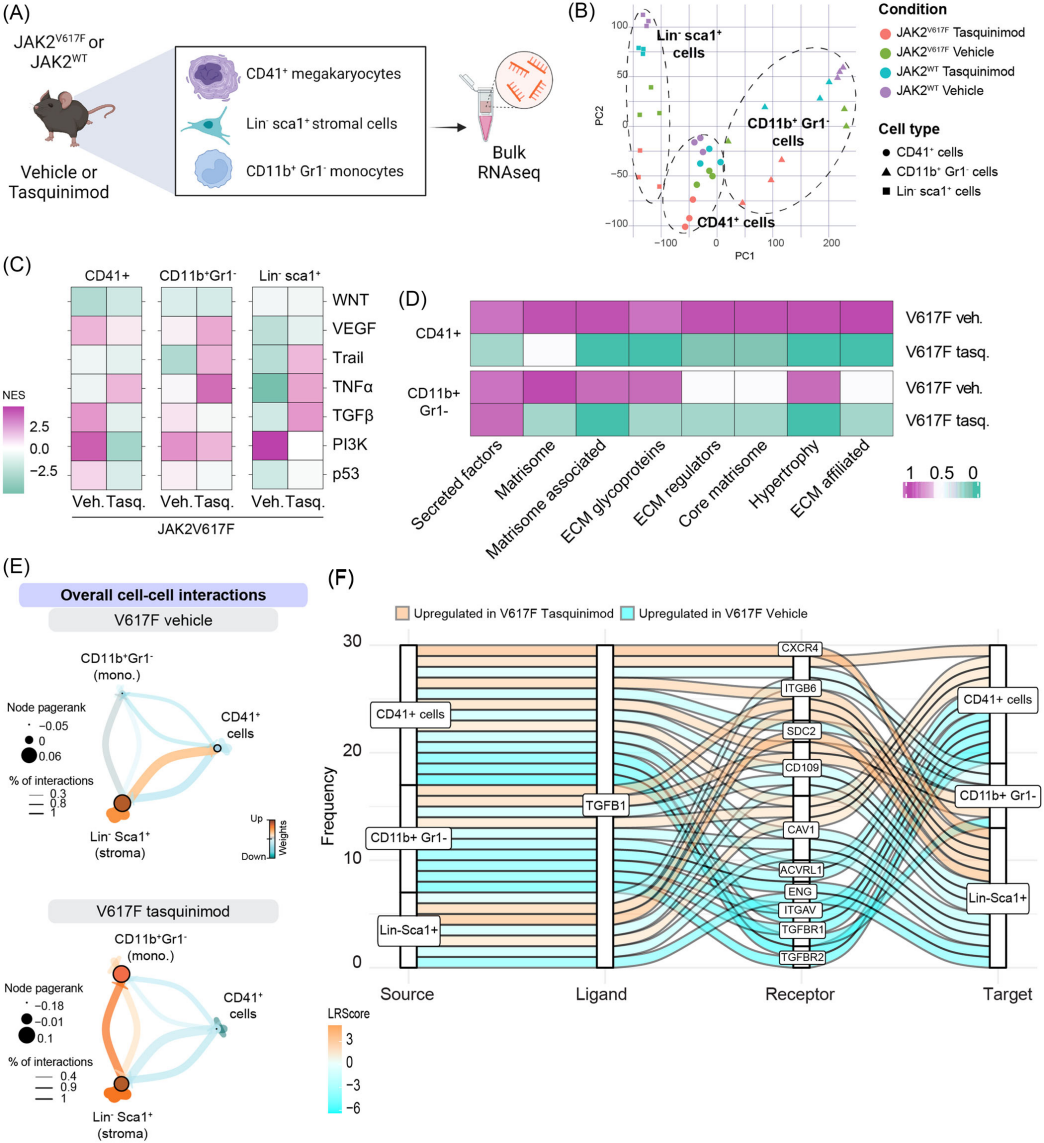
**S100A9‐inhibition using tasquinimod reveals novel transcriptome reprogramming and reversal of TGFβ activation. (A)** Experimental scheme of RNAseq sorting strategy for JAK2^WT^ or JAK2^V617F^ CD41^+^, CD11b^+^Gr1^−^, and Lin^−^Sca1^+^ cells, either treated with vehicle or tasquinimod (*n* = 3 mice/group/cell population). **(B)** Principal component analysis of JAK2^WT^ or JAK2^V617F^ CD41^+^, CD11b^+^Gr1^−^, and Lin^−^Sca1^+^ cells, either treated with vehicle or tasquinimod. **(C)** Heatmap representation of PROGENy analysis in CD41^+^, CD11b^+^Gr1^−^, and Lin^−^Sca1^+^ cells. **(D)** Heatmap representation of extracellular matrix (ECM)‐related Hallmark pathways in CD41^+^, CD11b^+^Gr1^−^, and Lin^−^Sca1^+^ cells, comparing JAK2^V617F^ vehicle versus JAK2^WT^ vehicle (labeled as “V617F veh”) and JAK2^V617F^ tasquinimod versus JAK2^V617F^ vehicle (labeled as “V617F Tasq”). **(E)** Overall cell‐to‐cell interactions between CD41^+^, CD11b^+^Gr1^−^, and Lin^−^Sca1^+^ cells. **(F)** Top 30 deregulated interactions mediated by TGFB1 between CD41^+^, CD11b^+^Gr1^−^, and Lin^−^Sca1^+^ cells. Interactions ordered based on the difference in mean LR expression between JAK2^V617F^ tasquinimod and JAK2^V617F^ vehicle.

We previously tested the S100A9‐inhibitor tasquinimod in the murine JAK2^V617F^ transplantation MF model, which normalized the myeloproliferation, extramedullary hematopoiesis, and prevented the development of BM fibrosis.^1^ We now sought to dissect which effect tasquinimod treatment has on both the mutant hematopoietic clone and on fibrosis‐driving stromal cells. We focused the analysis on megakaryocytes, stromal cells, and monocytes as we demonstrated that significant alarmin S100A8/S100A9 interactions existed between these cell types in human MF. We thus sort‐purified and performed RNA sequencing on GFP^+^CD41^+^ megakaryocytes, GFP^+^CD11b^+^Gr1^−^ monocytes, and Lin^−^Sca1^+^Pdgfra^+^ stromal cells from JAK2^V617F^‐driven MF (Figure S1A), from the time point at which mice treated with vehicle showed signs of fibrosis development, based on surrogate markers in blood (Figure 1A). Principal component analysis (PCA) showed distinct clustering of the three different cell types and a separation between the tasquinimod‐treated versus vehicle‐treated controls, specifically in the hematopoietic cell types (Figure 1B). Although the tasquinimod‐treated cells clustered apart from the controls, they were also distinct in their gene expression from WT cells (Figure S1B–E).

We performed a Pathway RespOnsive GENes (PROGENy)^6^ analysis to understand which signaling pathways are affected in (1) JAK2^V617F^ vehicle versus JAK2^WT^ vehicle (fibrosis vs. no fibrosis) and (2) JAK2^V617F^ tasquinimod versus JAK2^V617F^ vehicle (tasquinimod‐treated fibrosis vs. untreated fibrosis). We observed that tasquinimod lowered the TGFβ, PI3K, and p53 pathway activity in JAK2^V617F^ disease, all known players in MPN and MF, specifically in CD41^+^ megakaryocytes and CD11b^+^Gr1^−^ monocytes (Figure 1C). In Lin^−^Sca1^+^ stromal cells, PI3K pathway activity was also decreased upon tasquinimod treatment, whereas Trail, TNFα, and TGFβ activities were rather increased. Previously, we demonstrated that in advanced disease, fibrosis‐driving stromal cells are transcriptionally reprogrammed and characterized by downregulation of pathway activity of, for example, TNFα, WNT, and VEGF.^1^, ^2^ Thus, these data potentially can indicate the decreased transcriptional reprogramming in those cells upon tasquinimod treatment and an earlier state of fibrosis.

As TGFβ was downregulated in CD41^+^ megakaryocytes and CD11b^+^Gr1^−^ monocytes, we next asked how extracellular matrix (ECM)‐associated proteins, such as secreted factors, matrisome, and ECM regulators, are changed with tasquinimod treatment in JAK2^V617F^ MF. We used published gene signatures (NABA) and observed a significant downregulation of ECM secreted factors, ECM glycoproteins, ECM‐regulated, and ‐affiliated proteins in CD41^+^ megakaryocytes and CD11b^+^Gr1^−^ monocytes showing a direct effect of tasquinimod on TGFβ signaling and associated ECM remodeling on the hematopoietic clone (Figure 1D).

We therefore hypothesized that tasquinimod acts on the TGFβ‐driven cellular crosstalk between mutant hematopoietic cells and fibrosis‐driving stromal cells. By applying CrossTalkeR^7^ on the sort‐purified populations, we dissected how tasquinimod treatment affects their communication. In untreated JAK2^V617F^‐driven fibrosis, the strongest overall interaction was determined between stromal cells and megakaryocytes (Figure 1E). Upon treatment with tasquinimod, this interaction was downregulated, and the main interaction occurred between Lin^−^Sca1^+^ stromal cells and monocytes, in line with more inflammatory pathways (Figure 1E). As the pathway activity of TGFβ was downregulated in CD41^+^ megakaryocytes and CD11b^+^Gr1^−^ monocytes, we next interrogated receptor–ligand interactions affecting TGFb1. In these cells, interactions of TGFb1 with TGFbR1 and R2, Integrin alpha V (Itgav), Endoglin (Eng), and CD109, all playing a role in ECM anchoring, cell migration, and thus fibrosis, were decreased (Figure 1F). In summary, these results demonstrate that tasquinimod treatment affects pro‐fibrotic TGFβ interactions and signaling in monocytes and megakaryocytes, thereby decreasing the fibrotic activation of stromal cells.

### Tasquinimod treatment induces apoptosis in mutant hematopoietic cells and reduces all S100A8/S100A9 interactions between fibrosis‐inducing and ‐driving cells

**Figure 2:**
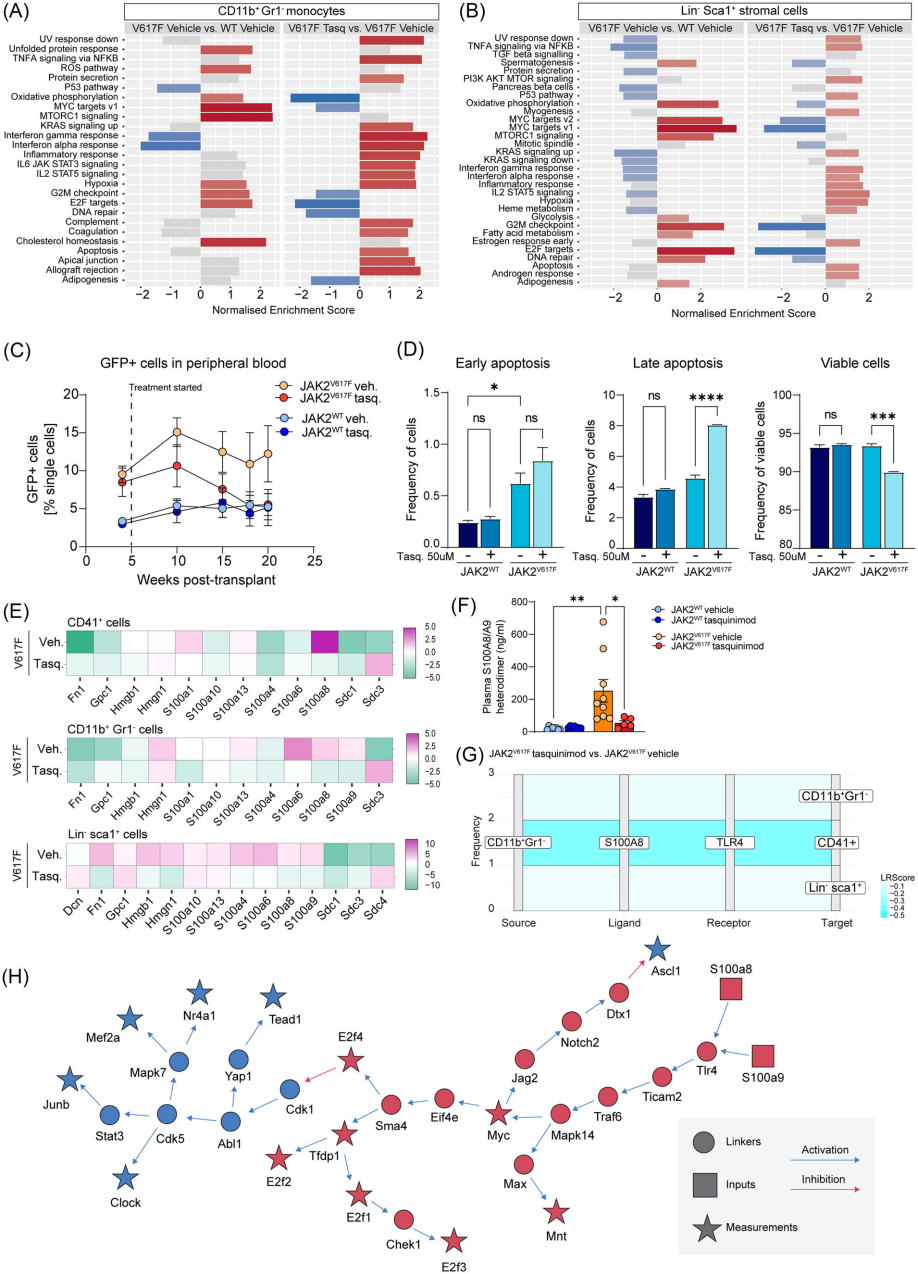
**Tasquinimod treatment abrogates Myc activation and induces JAK2** ^**V617F**^ **‐specific apoptosis. (A)** Hallmark gene set enrichment analysis (GSEA) of Gr1^−^CD11b^+^ monocytes and **(B)** Lin^−^Sca1^+^ stromal cells, comparing JAK2V617F vehicle versus JAK2WT vehicle (labeled as “V617F vehicle”) and JAK2V617F tasquinimod versus JAK2V617F vehicle (labeled as “V617F tasquinimod”). **(C)** Frequency of GFP^+^ cells in peripheral blood over time. **(D)** Flow analysis of early‐stage cell apoptosis (Annexin V^+^), and late‐stage apoptosis (Annexin V^+^ 7‐AAD^+^) in JAK2^WT^‐HoxB8‐Flt3 or JAK2^V617F^‐HoxB8‐Flt3 cells treated with 50 µM tasquinimod or DMSO control for 24 h. **(E)** Heatmaps of differential analysis focused on damage‐associated molecular pattern (DAMP)‐associated genes. All comparisons are JAK2^V617F^ vehicle versus JAK2^WT^ vehicle (labeled as “V617F vehicle”) and JAK2^V617F^ tasquinimod versus JAK2^V617F^ vehicle (labeled as “V617F tasquinimod”). **(F)** Enzyme‐linked immunosorbent assay (ELISA) analysis of the S100A8/S100A9 heterodimer on murine plasma samples from JAK2^WT^ or JAK2^V617F^ animals, either treated with vehicle or tasquinimod drinking water (*n* = 7–10/group). **(G)** Sankey plot showing S100a8‐Tlr4 ligand–receptor interactions in Gr1^−^CD11b^+^ monocytes. **(H)** CARNIVAL‐based pathway inference of the effect of S100a8/S100a9 binding to Tlr4.

We performed gene set enrichment analysis (GSEA) using Hallmark gene sets^8^ on all three cell populations to further dissect the effect of tasquinimod on hematopoietic and non‐hematopoietic cells. In all three cell types, we observed four global patterns comparing untreated JAK2^V617F^ disease (JAK2^V617F^ vehicle compared to WT vehicle) to tasquinimod‐treated disease JAK2^V617F^ disease: (1) downregulation of pro‐proliferative pathways (E2F targets, G2M checkpoint), (2) downregulation of MYC targets and MTORC signaling, (3) upregulation of inflammatory signaling (Interferon γ and α response; IL6 signaling), and (4) increased apoptosis. The increased inflammatory signaling might be a reflection of an increased monocyte and macrophage response upon treatment. Decreased pro‐proliferative signaling and increased apoptosis might also suggest a direct inhibitory effect of tasquinimod on mutant cells. In line with this, we observed an expansion of JAK2^V617F^‐GFP^+^ cells in untreated JAK2^V617F^ mice, which is abrogated with tasquinimod treatment (Figure 2C). To confirm that tasquinimod affects apoptosis in JAK2^V617F^ but not normal hematopoietic stem and progenitor cells, we used flow cytometry to measure apoptosis in HoxB8‐Flt3 cells, which either carry the JAK2^WT^ or JAK2^V617F^ variant. Critically, tasquinimod specifically induces early and late apoptosis in JAK2^V617F^‐HoxB8‐Flt3 cells, but not in JAK2^WT^‐HoxB8‐Flt3 cells (Figure 2D). Moreover, we treated the SET‐2 human megakaryoblastic cell line, which harbors the JAK2^V617F^ mutation, with either 50 µM of tasquinimod or vehicle for a maximum of 48 h and performed quantitative real‐time polymerase chain reaction (qRT‐PCR) on a panel of genes related to canonical and noncanonical TGFb signaling (Figure S2C). Overall, tasquinimod treatment downregulates canonical and noncanonical TGF‐b signaling, specifically PI3K/AKT/mTOR (PAM) related genes.

Damage‐associated molecular patterns (DAMPs) are molecules released or exposed by dead, dying, injured, or stressed non‐apoptotic cells, with multiple roles in inflammation, immunity, and cancer.^9^ As we observed increased apoptosis upon tasquinimod treatment, and S100A8/S100A9 are DAMPs, we next looked specifically into DAMP‐associated genes. The upregulation of DAMP‐associated genes was most pronounced in monocytes and stromal cells upon tasquinimod treatment, affecting mostly the syndecans Sdc3 and Sdc4 (Figure 2E). Critically, S100a8 and S100a9 were downregulated in all three cell types, confirming the direct effect of tasquinimod on these alarmins. At the functional level, we observed a complete normalization of the S100a8/S100a9 heterodimer (calprotectin) levels in plasma of JAK2^V617F^ mice treated with tasquinimod at the time of sacrifice (Figure 2F), correlating with a downregulation of the S100a8 and S100a9 genes in CD41^+^ cells, CD11b^+^Gr1^−^ monocytes, and Lin^−^Sca1^+^ stromal cells (Figure 2E). We stained femoral sections and showed a significant reduction in the frequency of S100A8+ cells in JAK2^V617F^ mice treated with tasquinimod, compared to the vehicle‐treated JAK2^V617F^ group (Figure S2A,B). In line with this, receptor–ligand interactions focusing on S100A8 demonstrated downregulation of all S100a8‐Tlr4 mediated interactions of monocytes with monocytes, megakaryocytes, and stromal cells, demonstrating that the S100a8/S100a9‐Tlr4 axis is the most significantly affected pathway in tasquinimod‐treated JAK2^V617F^ MPN associated with fibrosis (Figure 2G).

Based on the altered transcription factor (TF) activity we observed based on DoRothEA analysis,^10^, ^11^ we next used CARNIVAL^12^ with a prior knowledge network from OmniPath^13^ to infer a signaling network between an inhibited S100a8/a9‐Tlr4 interaction and the downstream estimated changes in TF activity (Figure 2H). Interestingly, in the putative signaling network, Myc acts as a central node. Downstream of Myc are the E2f family of TFs, namely E2f1, E2f2, E2f3, and E2f4, as well as Tfdp1. These are all TFs whose activity is estimated to be upregulated in fibrosis and downregulated after tasquinimod treatment. In fact, the estimated downregulation of Myc, Tfdp1, and E2f TF activities under tasquinimod is consistently seen across the three cell types. Thus, the DoRothEA and CARNIVAL analysis suggest a mechanistic hypothesis as to the signaling path through which tasquinimod treatment reverses the JAK2^V617F^‐induced deregulation of TFs starting with binding of the alarmins S100a8/S100a9 to Tlr4 regulating proliferation and apoptosis through Myc and E2f and suggesting a Myc‐Jag2‐Notch2 link that can be associated with remodeling of the tumor microenvironment.^14^

As the transcriptome analysis emphasized the central role for alarmins S100a8/S100a9 in the tasquinimod‐mediated effect on fibrosis and the MPN phenotype, we next sought to systematically dissect the effect of S100a9 (and S100a8) knockout (1) in the stroma and (2) in hematopoietic cells on the fibrotic transformation. We specifically hypothesized that the alarmin crosstalk originates in hematopoietic cells/monocytes and is communicated to stromal cells.

### S100A9 knockout in stromal cells is not sufficient to prevent BM fibrosis and MPN development

**Figure 3:**
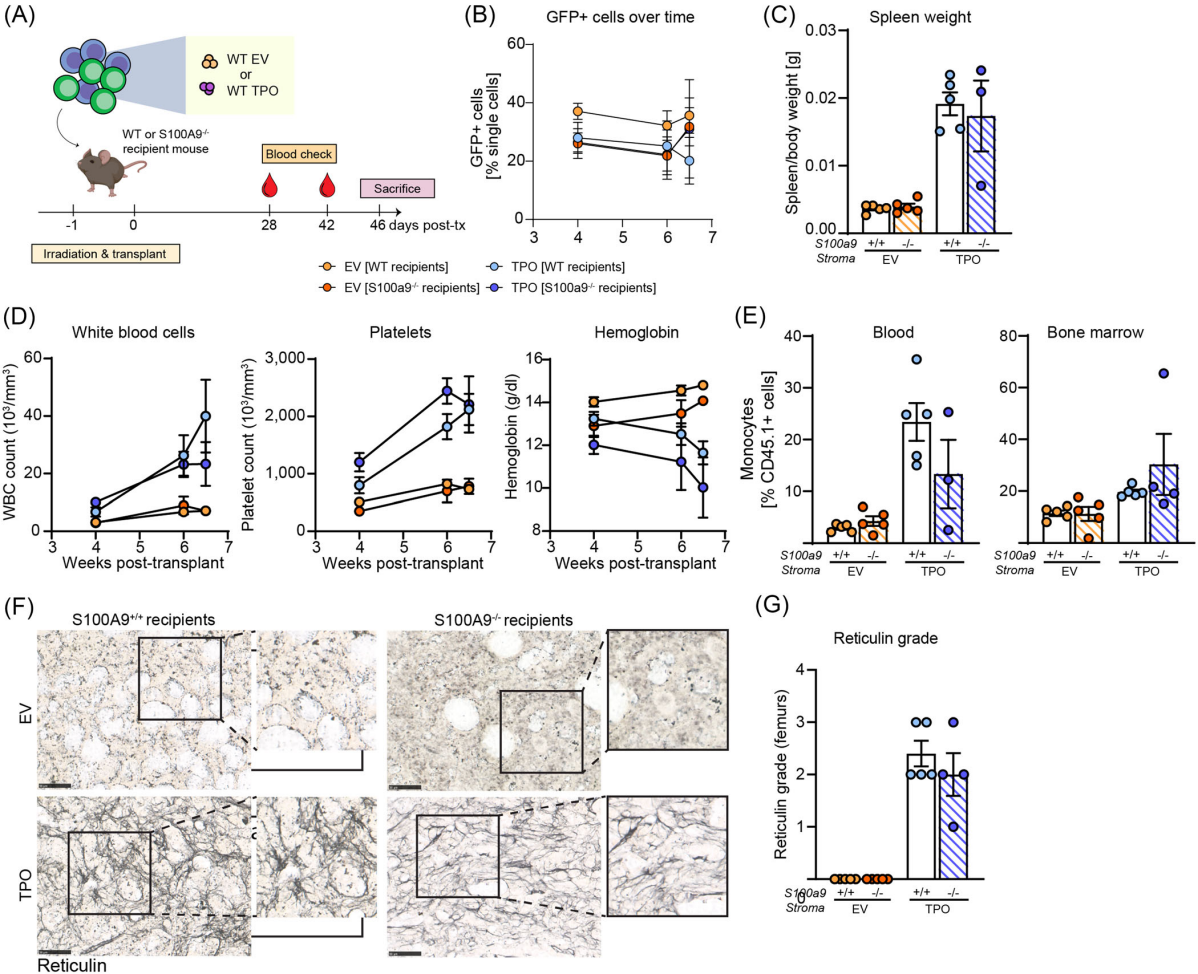
**Absence of S100a9 in stroma does not ameliorate bone marrow (BM) fibrosis in myeloproliferative neoplasm (MPN). (A)** Schematic representation of transplantation of WT ckit^+^ cells in a model of TPO‐induced fibrosis into either WT or S100a9^−/−^ recipient mice (*n* = 4–5 mice/group). **(B)** Frequency of TPO‐GFP^+^ or EV‐GFP^+^ cells in peripheral blood over the course of the experiment. **(C)** Spleen‐to‐bodyweight ratio (g) in recipient animals. **(D)** WBC, platelet, and hemoglobin parameters in weeks posttransplant. **(E)** Frequency of monocytes in peripheral blood and bone marrow at 6.5 weeks posttransplant in WT and S100A9^−/−^ recipient animals. **(F)** Representative images of reticulin staining and **(G)** myelofibrosis grading based on reticulin staining. Scale bar, 100 μm. Data are shown as mean ± standard error of the mean, one‐way analysis of variance followed by Tukey's post hoc test. *P < 0.05, **P < 0.01, ***P < 0.001, and ****P < 0.0001. ns, not significant.

As we previously identified increased expression of S100a8/S100a9 in the fibrosis‐driving MSCs,^1^ we first analyzed the effect of the absence of S100a8/S100a9 on the MPN phenotype and fibrosis. Thus, we transplanted S100a9^−/−^ or WT mice as recipients of either EV‐ or TPO‐transduced ckit^+^ HSPCs (Figure 3A). Transduced GFP^+^ cells remained stable and consistent between groups after transplant until the end of the experiment (Figure 3B). Splenomegaly, which is indicative of extramedullary hematopoiesis, was not ameliorated in S100A9^−/−^ recipient mice (Figures 3C and S3A), suggesting strong BM myeloproliferation and disease progression in groups with stromal S100a9 knockout similar to WT recipients. Blood values such as WBCs and platelets were comparable in the absence and presence of S100a9 in the TPO‐induced MPN and BM fibrosis model. In line with these findings, hemoglobin in S100A9^−/−^ recipients decreased at a similar rate to WT recipients in TPO‐induced disease (Figure 3D). Moreover, monocytosis, a common feature in PMF, was not ameliorated in S100A9^−/−^ recipients in either peripheral blood or BM (Figure 3E). The absence of S100a9 in the stroma did not have an effect on the development of fibrosis in the TPO‐induced MPN/fibrosis model, and megakaryocyte abnormalities were comparable in S100a9 and WT recipients (Figure S3B). We thus concluded that the absence of S100a9 in the BM stroma is not sufficient to improve BM fibrosis in MPN.

### Inhibition of the hematopoietic‐driven S100A9 crosstalk ameliorates BM fibrosis in MPN

**Figure 4:**
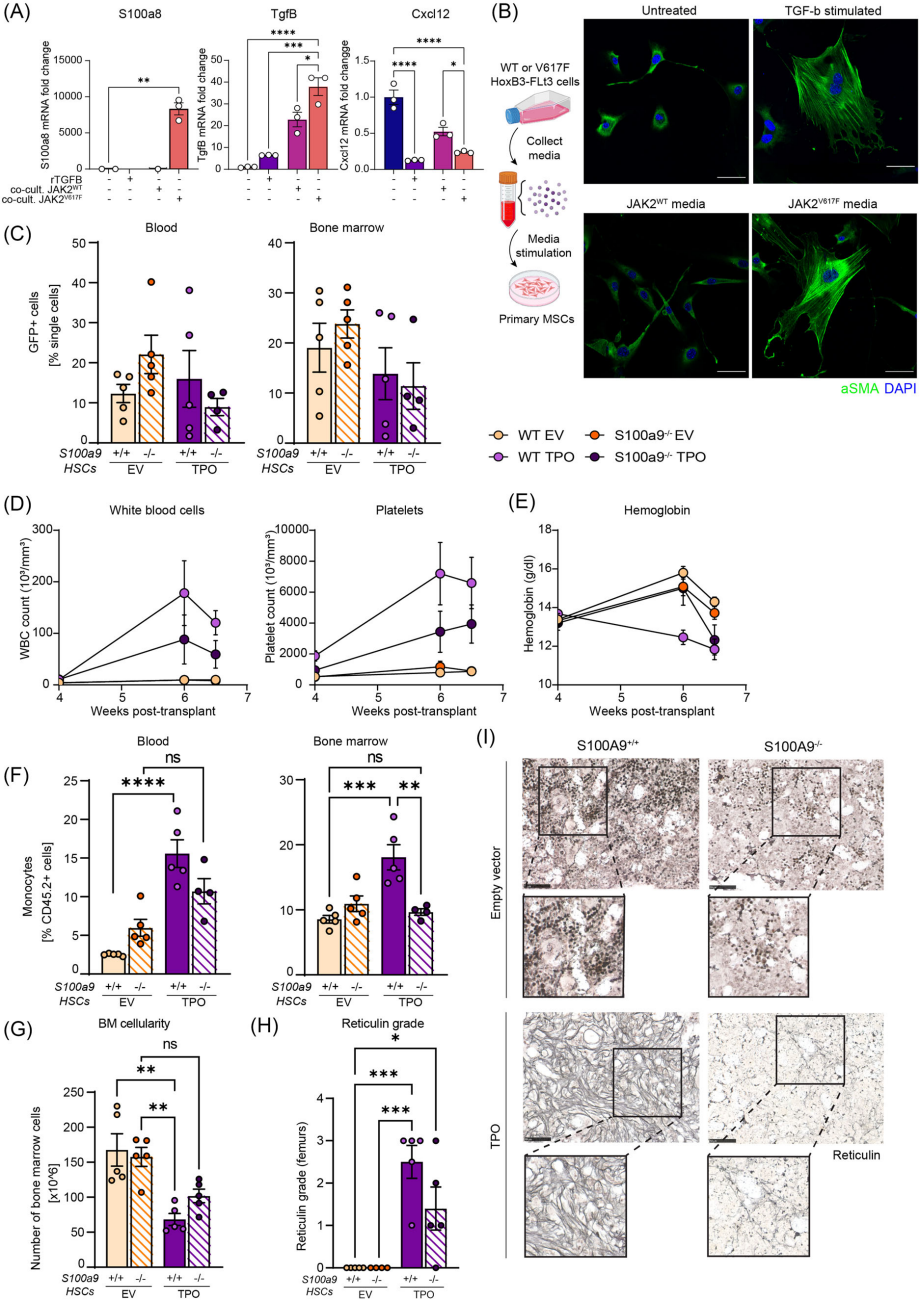
**Absence of S100A9 in hematopoietic stem cells (HSCs) ameliorates anemia, reduces monocytosis, and reduces bone marrow (BM) fibrosis in TPO‐driven myeloproliferative neoplasm (MPN). (A)** Quantitative polymerase chain reaction of S100a8, Tgfβ, and Cxcl12 in primary murine stromal cells (MSCs) cocultured with JAK2^WT^‐HoxB8‐Flt3, JAK2^V617F^‐HoxB8‐Flt3 cells, Tgfβ‐stimulated or untreated. *n* = 3/group. **(B)** Representative images of αSMA staining of primary murine stromal cells were cultured with 72‐h‐old media from JAK2^WT^‐HoxB8‐Flt3 or JAK2^V617F^‐HoxB8‐Flt3 cells, untreated or Tgfβ‐stimulated. **(C)** Frequency of TPO‐GFP^+^ or EV‐GFP^+^ cells in peripheral blood and BM at 6.5 weeks posttransplant (sacrifice). **(D)** WBC and platelet parameters in weeks posttransplant. **(E)** Hemoglobin levels in weeks posttransplant. **(F)** Frequency of monocytes in peripheral blood and BM at 6.5 weeks posttransplant. **(G)** Number of total BM cells isolated from 2 femurs, 2 tibias, and 2 pelvic bones (*n* = 5/group). **(H)** Myelofibrosis grading based on reticulin staining and **(I)** representative images of the BM reticulin staining. Scale bar, 100 μm. Data are shown as mean ± standard error of the mean, one‐way analysis of variance followed by Tukey's post hoc test. *P < 0.05, **P < 0.01, ***P < 0.001, and ****P < 0.0001. ns, not significant.

Next, we tested the hypothesis that the crosstalk of hematopoietic cells to stromal cells is crucial to induce S100a8/S100a9 expression in stromal cells and thus needs to be inhibited. To functionally validate that mutant hematopoietic cells can induce S100a9 in the stroma, we used coculture experiments between HoxB8‐Flt3 cells, which either carry the JAK2^WT^ or JAK2^V617F^ variant, and primary MSCs, and demonstrated that the direct interaction between JAK2^V617F^‐mutated cells and MSCs upregulates *S100a8* in stromal cells but not the addition of Tgfβ alone. TGFβ addition and coculture with JAK2^V617F^ mutant cells increased the expression of *Tgfβ* in stromal cells, and downregulated *Cxcl12 as* a key factor in hematopoiesis support (Figure 4A). Interestingly, supernatant collected from JAK2^V617F^‐HoxB8‐Flt3 cells applied to primary murine stromal cells alone already led to their activation and development of αSMA fibers, similar to TGF‐β stimulation (Figure 4B).

We combined our murine model of TPO overexpression‐induced MPN/myelofibrosis with a knockout of *S100a9* in HSPCs, and transplanted lethally irradiated WT recipients with either WT (here referred to as *S100a9* ^*+/+*^) or *S100a9* ^−/−^ ckit^+^ HSPCs overexpressing TPO or its EV control (Figure S4A). We confirmed that transduction efficiency between WT TPO and S100A9^−/−^ TPO cells was similar before transplant (Figure S4B), and after engraftment in peripheral blood and BM (Figure 4C) with >90% peripheral blood chimerism (Figure S4C). The absence of *S100a9* in HSPCs in TPO induced MF led to a reduction of WBC and platelet counts compared to HSPCs expression normal S100a9 levels, indicating an improvement of the MPN phenotype (Figure 4D). Additionally, we observed a slower decrease in hemoglobin levels in peripheral blood in the setting of S100a9 knockout in hematopoietic cells (Figure 4E), suggesting an amelioration of the MPN phenotype and also fibrosis, which is associated with increasing cytopenias, in particular anemia. Splenomegaly, which is indicative of extramedullary hematopoiesis, was ameliorated with the loss of *S100a9* in hematopoietic cells. We showed a reduction of monocyte counts in both peripheral blood and BM in TPO mice in the absence of S100a9 in hematopoietic cells (Figure 4F). Importantly, the drop in BM cellularity associated with disease progression was ameliorated in mice that lacked *S100a9* in HSPCs (Figure 4G), concurrent with a significant reduction in reticulin fibrosis (Figure 4H,I).

**Figure 5:**
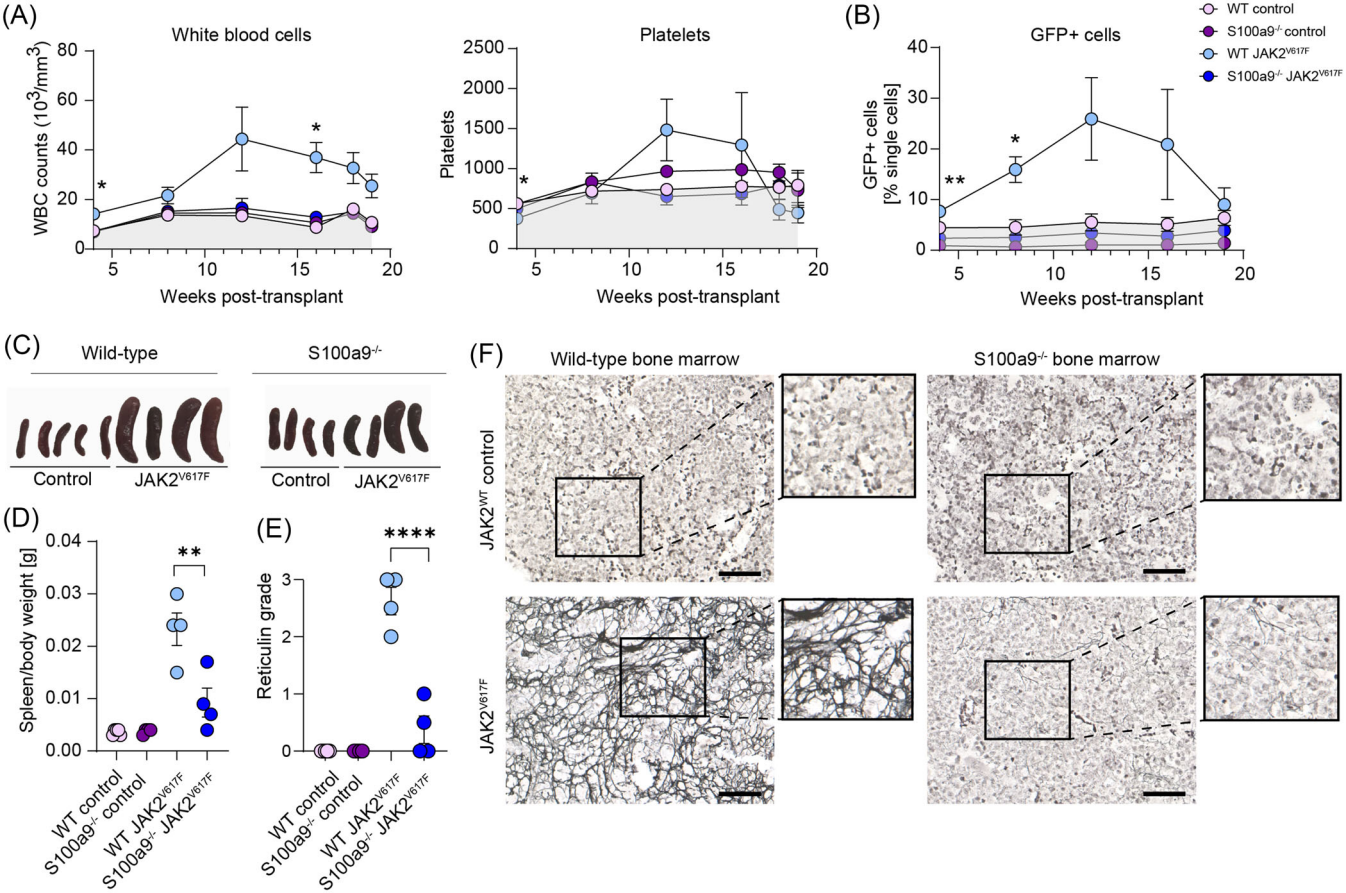
**Genetic knockout of hematopoietic S100A9 in the JAK2** ^**V617F**^ **‐retroviral model ameliorates the myeloproliferative neoplasm (MPN) phenotype and normalizes bone marrow (BM) fibrosis. (A)** WBC and platelet count in WT or S100A9^−/−^ JAK2^V617F^‐ or JAK2^WT^ mice in weeks posttransplant. *P < 0.05, refers to comparisons between WT control and WT JAK2V617F, and WT JAK2^V617F^ and S100A9^−/−^ JAK2^V617F^. **(B)** Frequency of JAK2^V617F^‐GFP^+^ or JAK2^WT^‐GFP^+^ cells in peripheral blood obtained over the course of the experiment. **P < 0.01, refers to comparisons between WT control and WT JAK2V617F, and WT JAK2^V617F^ and S100A9^−/−^ JAK2^V617F^. **(C)** Images of spleens and **(D)** spleen‐to‐bodyweight ratio in WT or S100A9^−/−^ JAK2^V617F^‐ or JAK2^WT^ mice. **(E)** Myelofibrosis grading of femoral BM and **(F)** representative images of reticulin staining. Data are shown as mean ± standard error of the mean, *n* = 4–5 per group, one‐way analysis of variance followed by Tukey's post hoc test. *P < 0.05, **P < 0.01, ***P < 0.001, and ****P < 0.0001. ns, not significant. Scale bar: 50 µm.

As we observed a significant improvement in BM fibrosis, splenomegaly, and monocytosis in the TPO‐induced PMF model, a robust model for MPN associated with fibrosis (Figure 3), we next sought to validate these findings in a JAK2^V617F^‐induced murine model of MPN/PMF as a highly patient‐relevant model. We transplanted lethally irradiated WT recipient mice with either WT or S100A9^−/−^ ckit+ HSPCs, transduced with the JAK2^WT^ or JAK2^V617F^ retroviral vectors. Impressively, in the JAK2^V617F^‐induced PMF model, WBCs and platelets in the S100a9^−/−^ group were completely normalized comparable to WT EV levels in the JAK2^V617F^ setting (Figure 5A). The absence of S100a9 in HSPCs inhibited the expansion of JAK2^V617F^ mutant cells (GFP^+^ cells) that is typically seen in disease (Figure 5B). Splenomegaly was significantly reduced in the absence of S100a9 in HSPCs (Figure 5C,D), and we observed an almost complete prevention of BM fibrosis development in femurs compared to the WT JAK2^V617F^ setting (Figure 5E,F).

### The absence of S100a9 in JAK2^V617F^‐driven MPN downregulates pro‐inflammatory and pro‐fibrotic pathways in monocytes and stromal cells

**Figure 6:**
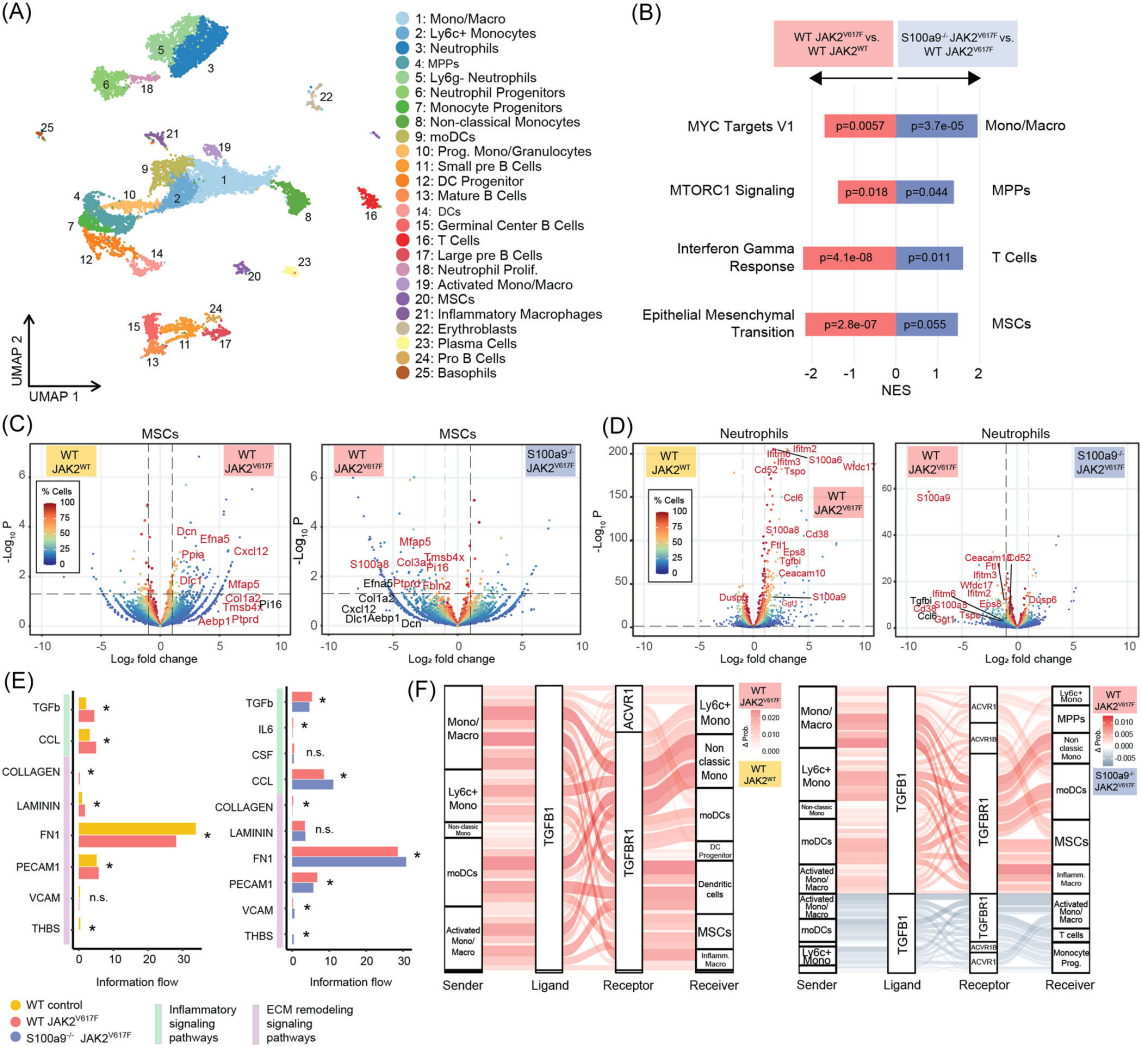
**scRNA sequencing reveals that loss of S100a9 in JAK2V617F‐driven myeloproliferative neoplasm (MPN) reduces pro‐inflammatory and pro‐fibrotic signaling in monocytes and stromal cells. (A)** UMAP representation of 13,491 cells based on the integration of three libraries (*n* = 3 mice each per group: WT JAK2^WT^, WT JAK2^V617F^, and S100A9^−/−^ JAK2^V617F^). **(B)** Representative plot of the Hallmark gene set enrichment analysis (GSEA) for specific cell types showing rescued pathways by S100a9^−/−^ JAK2^V617F^ compared to WT JAK2^V617F^. **(C, D)** Volcano plots displaying differentially expressed genes between conditions in mesenchymal stromal cells (MSCs) or neutrophils. Genes with significant differential expression (P < 0.05 and log2 fold change >1) are located above threshold lines. Genes of interest appear labeled in red when significant and in black when P > 0.05. Dot color is given by the % cells expressing the gene. **(E)** Bar plot showing cell–cell interaction pathway differences in the overall information flow within the inferred networks between conditions. The star on top indicates significance after performing a paired Wilcoxon test. Pathways shown are grouped in inflammatory and extracellular matrix (ECM) remodeling signaling pathways. **(F)** Sankey plot showing all differential interactions mediated by TGFB1 between monocyte‐related cells and the rest of the cells in the data set. Only interactions with a differential interaction score greater than 0.001 are shown. The width of each flow corresponds to the magnitude of the differential interaction score.

To understand the effect of knockout of S100A9 in hematopoietic cells on the pro‐fibrotic immune cell‐stromal crosstalk in JAK2^V617F^‐induced MPN, and to compare the results to the treatment with tasquinimod, we performed single‐cell RNA sequencing (scRNAseq;10x Genomics platform). To enrich for stromal cells, we performed a lineage‐depletion of the whole BM from the JAK2^V617F^ experiment (Figure 5). We pooled the lineage negative fraction from three mice from each of the WT JAK2^WT^, WT JAK2^V617F^, and S100A9^−/−^ JAK2^V617F^ groups and supplemented lineage positive cells to enrich for immune cells. Clustering after batch correction and integration identified 25 clusters including immune cell subtypes enriched for myeloid cells and their progenitors, lymphoid cells, and a cluster of MSCs (Figures 6A and S5A). We recovered a total of 13,491 cells, and the three different experimental conditions were evenly distributed in the clusters (Figure S5B,C). We sought to understand if the absence of S100A9 in hematopoietic cells would have a similar effect as tasquinimod. We thus performed Hallmark GSEA and observed that Myc targets, MTORC1 signaling, interferon gamma response and the epithelial–mesenchymal transition (EMT) pathway are downregulated in S100A9^−/−^ JAK2^V617F^ compared to WT JAK2^V617F^ in monocyte/macrophages, MPPs, T cells and stromal cells, respectively, in line with findings after tasquinimod treatment (compare Figure 2). In particular, the gene set “EMT” was of particular interest to us as it entails genes responsible for the fibrotic transformation. We thus next focused transcriptional changes in MSCs as the drivers of BM fibrosis.^1^ In WT JAK2^V617F^, MSCs significantly upregulate decorin (DCN) as a marker for myofibroblasts, as well as collagens. In the absence of S100a9, we observed a strong downregulation of genes related to the fibrotic transformation in the S100a9^−/−^ JAK2^V617F^ condition, such as Dcn, Fbln2, Col1a2, Col3a1, as well as S100A8 (Figure 6C), in line with the significant reduction of BM fibrosis.

We wondered which cell types are mostly affected by the JAK2^V617F^ mutation in our data set and also by S100a9 knockout. Interestingly, not only monocyte progenitors, Ly6c+ monocytes, and their progenitors but also granulocytes and neutrophils showed the most prominent transcriptional changes in JAK2^V617F^, and exactly these populations and monocytes were mostly affected by the S100a9 knockout (Figure S5D). Further analyses of these cell populations pointed out that neutrophils in JAK2^V617F^ show a significant upregulation of not only inflammatory‐related genes (Ifitm2, Ifitm3, Ifitm6, Ccl6, S100a8, and S100a9) but also ECM‐related genes (Ceacam10, Eps8) compared to the WT control group. Importantly, almost all of these genes were normalized in the S100A9^−/−^ JAK2^V617F^ condition (Figure 6D). This highlights that S100A9 knockout in hematopoietic cells not only reduces both the fibrotic transformation of MSCs but also significantly reduces the inflammation pathognomonic for MPN in myeloid cells. As this indicates that S100a9 indeed modifies the cellular crosstalk between hematopoietic (myeloid) and MSCs, we further interrogated cell–cell interactions across the data set. In WT JAK2^V617F^, fibrosis‐related crosstalk was significantly upregulated in comparison to the control, including TGFb‐, collagen‐, and PECAM1‐driven crosstalk (Figure 6E). In line with the reduced activation of stromal cells, the communication in these pathways was significantly reduced in the absence of S100a9 in JAK2^V617F^‐driven disease. Looking more specifically into the TGFb1 ligand–receptor pairs, we observed an upregulation of the TGFb1–TGFbR1 interactions between monocyte subtypes and MSCs in JAK2^V617F^‐mediated MPN compared to control. The absence of S100A9 specifically downregulated the TGFb1‐driven monocyte crosstalk (Figure 6F).

In conclusion, the data demonstrate that S100a9 knockout downregulates ECM remodeling and inflammatory signals in JAK2^V617F^‐driven disease, which are central mechanisms in the disease pathogenesis.

## DISCUSSION

Our research adds to the accumulating evidence highlighting inflammation, particularly from the hematopoietic compartment, as a central factor in the pathogenesis of fibrosis and associated cytopenias in MPNs. Inflammation originating from the hematopoietic malignant clone is a pivotal driver of BM fibrosis. Our study specifically identifies the S100A8/S100A9 heterodimer as a key factor in this process. These proteins, expressed by the malignant hematopoietic cells, contribute significantly to the fibrotic environment within the BM. This aligns with existing literature, which underscores the role of inflammatory signaling in the progression of MPN and associated BM fibrosis.^1^, ^15^, ^16^

Our previous research demonstrated that tasquinimod effectively ameliorates the MPN phenotype, reducing splenomegaly and normalizing fibrosis in a JAK2^V617F^‐driven preclinical model of MPN. However, the precise mechanisms by which tasquinimod influences BM fibrosis remained unclear until our current investigation. Using bulk RNA sequencing, we have elucidated that tasquinimod exerts its primary effects on the malignant, JAK2^V617F^‐mutant hematopoietic clone, particularly on megakaryocytes and monocytes. The analysis revealed significant downregulation of pro‐proliferative pathways, such as E2F targets and the G2M checkpoint, alongside reduced MYC targets and mTORC signaling. TGF‐β‐driven ECM remodeling can be mediated by mTORC1, which promotes collagen synthesis, and mTORC2, which facilitates EMT and cell invasion. MYC's role in ECM regulation is less well defined but may interact with TGF‐β signaling to regulate ECM dynamics.

Concurrently, there was an upregulation of inflammatory signaling pathways, including the interferon gamma and alpha response, and IL6 signaling, coupled with increased apoptosis within the malignant clone. Notably, tasquinimod induced apoptosis in JAK2V617F cells, both in vitro and in vivo, corroborating similar findings in multiple myeloma and acute myeloid leukemia where tasquinimod, especially in combination with venetoclax, led to reduced MYC targets, cell cycle regulators, and mTORC1 signaling.^17^ Additionally, a MYC‐S100A9 circuit was recently established in the context of triple negative MPN that provokes a complex network of inflammatory signaling that involves numerous hematopoietic cell types in the BM microenvironment.^18^ Our results suggest that tasquinimod also interferes with the MYC‐S100A9 circuit.

Our data indicate that tasquinimod reverses TGFβ‐driven fibrotic reprogramming in megakaryocytes and monocytes in the context of JAK2^V617F^‐driven MPN. This reversal is crucial for mitigating the pro‐fibrotic interactions and signaling in the BM, thereby decreasing the activation of stromal cells, which are instrumental in fibrosis. Further validating the role of the S100A8/S100A9 axis, our experiments involving the genetic ablation of S100A9 in the hematopoietic compartment, but not in the stromal cells, revealed a significant improvement in the MPN phenotype and normalization of BM fibrosis. This highlights the hematopoietic origin of the inflammatory signals driving fibrosis. It was recently demonstrated that chronic inflammation contributes to the increased fitness of mutant cells.^19^ Our results confirm that the malignant hematopoietic clone creates an inflammatory environment, which in turn alters stromal cells into fibrosis‐driving cells with decreased potential to support normal hematopoiesis.

To functionally validate the role of mutant hematopoietic cells in inducing S100A9 expression in the stroma, we conducted coculture experiments for 72 h. HoxB8‐Flt3 cells carrying either the JAK2^WT^ or JAK2^V617F^ variant were cocultured with primary MSCs. The results showed that direct interaction between JAK2^V617F^‐mutated cells and MSCs upregulated S100A8 in stromal cells, an effect not replicated by the addition of TGFβ alone. These results are interesting in the context of studies showing that TGFβ induces the fibrotic reprogramming of stromal cells but is not responsible for the hematopoietic niche reduction in MPN.^20^ Our results indicate that alarmins derived from the malignant clone contribute to the loss of hematopoiesis support in MPN by leading to downregulation of hematopoiesis‐supporting factors.

In summary, our findings elucidate the complex interplay between inflammation, hematopoietic malignancy, and fibrosis in MPN. These insights pave the way for potential therapeutic strategies targeting inflammatory signaling pathways in MPN to mitigate fibrosis and improve patient outcomes.

## MATERIALS AND METHODS

### Viral transduction

For retroviral and lentiviral transduction, lineage‐depleted or ckit^+^‐enriched cells from 8‐ to 12‐week‐old WT or S100a9^−/−^ mice were isolated by crushing compact bone and cells magnetically enriched for CD117 (ckit) by magnetic separation (Miltenyi Biotec). Ckit+ BM cells were pre‐stimulated for 24 h in StemSpan media (Stem Cell Technologies) supplemented by murine stem‐cell factor (m‐Scf, 50 ng/mL, Peprotech) and murine thrombopoietin (m‐Tpo, 50 ng/mL, Peprotech). Oncoretroviral vectors were pseudotyped with ecotropic envelope (pCL‐Eco, Addgene: 12371, a kind gift from Inder Verma) and produced using standard protocols. Retroviral transduction using pMIG‐JAK2^V617F^ or pMIG‐JAK2^WT^ was performed on retroNectin (Takara Bio)‐coated cell‐culture dishes loaded with unconcentrated virus. Cells were resuspended in virus‐containing medium in the presence of 4 μg/mL polybrene. Lentiviral particles overexpressing TPO‐GFP or EV‐GFP were produced by transient transfection with lentiviral plasmid together with psPAX2 and VSVG packaging plasmids using TransIT®‐LT1 Transfection Reagent (Mirus Bio). Lentiviral particles were concentrated by ultracentrifugation at 4°C. Lentivirus transductions were performed with concentrated lentiviral supernatant in the presence of 4 μg/mL polybrene at 37°C for a minimum of 24 h.

### Animal studies and induction of myelofibrosis by overexpression of TPO or JAK2^V617F^

PtprcaPepcb/BoyCrl (B6.SJL) mice were purchased from Charles River (the Netherlands) and maintained in specific‐pathogen‐free conditions. S100A9KO and S100A9 transgenic (S100A9Tg) mice on a C57BL/6 background have been described before^21^, ^22^ and were obtained from T. Vogl (Muenster, Germany). All mouse studies were conducted according to protocols approved by the Central Animal Committee (Centrale Commissie Dierproeven [CCD], the Netherlands) in accordance with legislation in the Netherlands (approval number: AVD1010020173387). Mice were maintained on a 12‐h light/dark cycle and were provided with water and standard mouse chow ad libitum.

WT or S100A9^−/−^ ckit^+^ cells transduced with JAK2^(V617F)^ or pMIG control retrovirus (control: JAK2^WT^), or TPO‐GFP or its control vector EV‐GFP, and 4–5 × 10^5^ cells isolated from three donors per group were transplanted into lethally irradiated (10.5 Gy) female B6.SJL recipients (*n* = 4–5 mice/group). For S100A9Tg experiments, whole BM cells from S100A9Tg mice or CD45.2^+^ WT (C57BL/6J) mice (*n* = 3 donors/group) were isolated from hind leg bones, and 2 × 10^6^ cells were transplanted into lethally irradiated (10.5 Gy) CD45.1^+^ B6.SJL recipient mice. For stromal knockout experiments, BM cells from CD45.1^+^ B6.SJL mice (*n* = 3 donors/group) were isolated from hind leg bones, enriched for CD117 using magnetic separation, and transduced using TPO‐GFP or EV‐GFP lentiviral vectors. 4–5 × 10^5^ cells were transplanted into lethally irradiated (10.5 Gy) C57BL/6J or S100A9^−/−^ recipient mice (*n* = 4–5 mice/group). Mice were randomly assigned to transplant groups and were sacrificed at 6.5 weeks posttransplant in the ThPO and 19 weeks post‐BM transplantation in the JAK2 experiment.

Blood was periodically collected from mice via submandibular bleeds into microtainer tubes coated with K_2_ ethylenediaminetetraacetic acid (EDTA) (Becton Dickinson, NJ, USA), and complete blood counts were performed on a Horiba SciI Vet abc Plus hematology system.

### Flow cytometry

BM cells were isolated by crushing the pelvis and hind leg bones in 2% fetal calf serum (FCS)/phosphate buffered saline (PBS) (GIBCO) and strained through a 70‐μm cell strainer. Whole BM was lysed at room temperature with red blood cell lysis buffer (BD Pharm Lyse) for 10 min and washed in 2% FCS/PBS. Cells were labeled with the following monoclonal, directly fluorochrome‐conjugated antibodies: anti‐mouse: Gr1 (ef450, BioLegend), Ter119 (ef450, BioLegend), CD3 (ef450, BioLegend), B220 (ef450, BioLegend), CD11b (ef450, BioLegend), ckit (APC, BioLegend), CD11b (APC, BioLegend), CD41 (PECy7, BioLegend), F4/80 (PECy7, BioLegend), Ter119 (APCCy7), CD48 (APCCy7, BioLegend), CD41 (APCCy7, BioLegend), Sca1 (PerCPCy5.5, BioLegend), CD45.2 (PerCPCy5.5, BioLegend), CD150 (PeCy7), CD45.2 (PE, BioLegend), and CD3 (PE, BioLegend). For flow cytometry, cells were stained with antibodies using a dilution of 1:100 in 2% FBS/PBS for 30 min on ice. All samples were analyzed by flow cytometry using a FACSFortessa or FACS Aria (BD Biosciences, San Jose, CA). Hoechst solution was added (1:10,000) to exclude dead cells in flow cytometric analyses, and data were analyzed using FlowJo software (Version 10, TreeStar Inc.).

### FACS‐staining and sorting of BM CD11b^+^Gr1^−^, Lin^−^Sca1^+^, and CD41^+^ cells

BM cells from tasquinimod‐treated (30 mg/kg/day, Active Biotech AB, Sweden) and vehicle‐treated JAK2V617F (or control JAK2WT) mice^1^ (*n* = 3 mice/group/cell population) were resuspended in 300 μL PBS/2% FCS and stained at 4°C for 20 min with the antibodies described below. After resuspension and addition of Hoechst (1:10,000), cells were sorted into 50 μL Dulbecco's modified eagle medium (DMEM)/10% FCS (BD Aria III) and directly mixed into Trizol LS. Monocytes were sorted as GFP^+^CD11b^+^Gr1^−^, megakaryocyte progenitors as GFP^+^ Gr1^−^ CD11b^−^ CD41^+^, and stromal cells were sorted as lin^−^ Sca1^+^PdgfrB^++^. Unstained cells were used as negative controls to define gating. All antibodies were purchased from BioLegend. The following fluorochrome‐conjugated antibodies were used: CD41‐PeCy7, CD11b‐PerCpCy5.5, Gr1‐APC‐Cy7, Ter119‐EF450, Sca1‐PE, and CD140b‐APC.

### RNA sequencing and bioinformatics

cDNA libraries of monocytes, sorted as GFP^+^CD11b^+^Gr1^−^, megakaryocyte progenitors as GFP^+^Gr1^−^ CD11b^−^ CD41^+^, and stromal cells were sorted as lin^−^ Sca1^+^PdgfrB^++^ were generated using the Smart‐Seq V4 ultra‐low input RNA kit (Clontech Laboratories) according to the manufacturer's instructions. Subsequently, amplified cDNA was further processed generating Illumina compatible sequence‐ready libraries using the Truseq Nano DNA sample prep guide (Illumina) that were pair‐end sequenced (2 × 75 cycles) on a Hiseq. 2500 platform (Illumina).

### Bulk RNA sequencing data analysis

Computational analysis of the bulk RNA‐seq data was performed in R. First, filtering was performed to remove lowly expressed genes. Counts were normalized and a variance‐stabilizing generalized log2 transformation performed using the vsn^23^ package. Differential expression analysis was performed using the limma^24^ package. GSEA was performed using the FGSEA^25^ package. Pathway enrichment was performed using the mouse PROGENy^6^, ^26^ resource, and scores were calculated using a weighted mean. TF activity estimation was performed with mouse DoRothEA resource,^10^, ^26^ and normalized enrichment scores were calculated using the VIPER aREA method.^27^ CARNIVAL^28^ was used to formulate an ILP optimization problem, solved using IBM CPLEX v12.10 under academic license, to predict an intracellular signaling network linking inhibition of the S100a8 and S100a9 activation of Tlr4 to the downstream estimated changes in TF activity between the JAK2^V167F^ tasquinimod and JAK2^V617F^ vehicle mouse models. OmniPath's^13^ signed and directed intracellular signaling network was used as a prior knowledge network as input for CARNIVAL.

### Single‐cell RNA sequencing of hematopoietic and stromal cells from S100A9KO JAK2^V617F^ in vivo experiment

At the time of sacrifice, femurs, tibiae, pelvises, and spines were dissected and cleaned of surrounding tissue as much as possible. The bones were crushed in 2% FCS/PBS using a mortar and pestle. Dissociated cells were filtered through a 70‐μm nylon cell sieve, spun down, lysed with PharmLyse RBC lysis buffer, and frozen viably. At the time of sequencing, cells were thawed as per normal procedure, and viability was measured. The cells were lineage‐depleted using biotinylated antibodies directed against lineages (CD5, CD45R, CD11b, Gr1, 7‐4, and Ter119) (Miltenyi Biotec) and additionally added CD45‐ and CD71‐biotin antibodies (BioLegend). After staining for 10 min at 4°C, the cells were washed and incubated with anti‐biotin beads (Miltenyi Biotec) for 15 min at 4°C before magnetic depletion using a MACS column (BD). We pooled viable cells from three mice from each of the WT JAK2^WT^, WT JAK2^V617F^, and S100A9^−/−^ JAK2^V617F^ groups and loaded 30,000 cells to obtain a fair representation and performed scRNAseq using the 10X Genomics platform v3.1.

Analysis was performed in R (v4.2.1) with Seurat^29^ (v5.1). Initial preprocessing removed ambient RNA contamination with SoupX^30^ (v1.6.2) using the auto‐estimation parameters. Then, cells with low QC parameters were filtered out (i.e., less than 200 distinct transcripts, or >20% mitochondrial counts); genes that were expressed in fewer than three cells were filtered out. Relative gene expression across cells was checked, and no abnormalities were observed.

Data were initially normalized for sequencing depth by dividing by the total number of unique molecular identifiers in every cell and then transformed to a log scale for each cell using the NormalizeData function. Scaling and PCA were performed; 50 principal components were calculated. Data were then integrated using reciprocal PCA. Data were inspected for doublets using DoubletFinder^31^ (v2.0.4), and cells labeled as doublets were removed. Cells were clustered using the shared nearest neighbor modularity optimization‐based clustering algorithm with resolutions from 0.1 to 1 in steps of 0.1. Optimal clustering resolution was chosen with the aid of clustree (v0.5.1). Lists containing differentially expressed genes within clusters were generated using Presto (v1.0) by computing auROC and Wilcoxon P‐value based on Gaussian approximation with a log fc threshold of 0.25. The output genes were used for manual cluster annotation based on the literature. Clusters that presented high expression of cell‐cycle‐related genes were labeled as “Proliferative.” Comparative analysis between conditions was done using pseudo‐bulk expression profiles in DESeq. 2 per cell type using a Wald test. DE lists per cell type were then used for pathway enrichment using fgsea (v1.24) using default settings. Hallmark database from the Human MSigDB Collections was used for pathway enrichment. Augur^32^ (v1.0.3) was used to identify cell populations that changed the most between conditions after performing binary and multiclass comparisons across experimental conditions. UMAP was used for the two‐dimensional representation of the data.

### Cell–cell communication analysis

Cell–cell communication analysis of the bulk RNAseq was done using R. First, RNA sequencing counts were normalized using count per million (CPM) normalization as described in the edgeR (v. 4.2) framework. Next, murine genes were mapped to human annotation using biomartR (v2.60), and the talklr tool (https://github.com/yuliangwang/talklr) was used to infer the ligand–receptor cell–cell communication for each condition. Finally, the talklr output was preprocessed, and a comparative analysis was performed using CrossTalkeR (v.1.3.2, https://github.com/CostaLab/CrossTalkeR).

For the scRNA data set, ligand–receptor analysis between groups and cell types was carried out with CellChat (v1.5). Briefly, the communication probability between cell types was calculated with the triMean method using all overexpressed signaling genes associated with each cell type with P‐value < 0.5 and FC > 0. The CellChatDB database was used for the ligand–receptor pairs and related pathways. All cell–cell pairs that were present in less than 10 cells were omitted.

### H&E, reticulin, and immunohistochemical staining of murine femurs

Murine organs were fixed in 4% paraformaldehyde for 24 h and transferred to 70% ethanol. Femurs were decalcified in 10% EDTA/Tris–HCl (pH 6.6) solution for 6 days, dehydrated, and paraffin‐embedded as standard. Hematoxylin and eosin (H&E) and reticulin staining were performed on 4‐μm sections according to routine protocols.

Immunohistological analysis of murine femur sections (4 μm) was performed using a primary antibody against S100A8 (Anti‐MRP8 antibody [EPR3554] S100A8, 1:200, Abcam 92331). Antigen retrieval was performed using citrate buffer in a conventional lab microwave (Vector, antigen unmasking solution). Sections were treated with 3% H_2_O_2_ and blocked with Avidin/biotin blocking kit (Vector), and incubated with primary antibody for 1 h at room temperature. Biotinylated monoclonal goat anti‐rabbit (Vector) was used as a secondary antibody for 30 min at room temperature. Slides were incubated with AB complex for 30 min at room temperature, washed, and incubated for a further 10 min with 3,3'‐diaminobenzidine substrate. Slides were stained with hematoxylin and mounted with a glass coverslip using DPX mountant (Sigma).

### Isolation of MSCs

MSCs were isolated from 8‐ to 10‐week‐old bigenic Gli1CreER;tdTomato mice after receiving 3 × 10 mg Tamoxifen p.o. administration or from 8‐ to 2‐week‐old WT mice. Compact bones were crushed and bone fragments were collected and digested with 10 mL collagenase 2 (1 mg/mL, Invitrogen) for 90 min at 37 °C in DMEM (GIBCO) supplemented with 10% FBS (GIBCO). The bone fragments were then washed with PBS (GIBCO) three times and moved to a 15‐cm cell‐culture dish. The bone chips were grown in alpha MEM (GlutaMAX, GIBCO), 20% MSC‐qualified FBS (GIBCO), 2% penicillin‐streptomycin (GIBCO), 1 ng/mL murine basic fibroblast growth factor (Peprotech), and 5 ng/mL murine epidermal growth factor (Peprotech).

### Generation and cell culture of JAK2V617F and JAK2WT Hoxb8‐FL progenitor cell lines

BM cell isolation was performed as described in the supplementary information. Isolated BM cells were resuspended at a concentration of 5 × 105 cells/mL in Iscove's modified Dulbecco's medium (IMDM) (GIBCO) with recombinant mouse IL‐3 (10 ng/mL), IL‐6 (20 ng/mL), and 50 ng/mL recombinant SCF (R&D system). After 2 days of cell culture, cells were seeded at a concentration of 2 × 10^5^/mL per well in a 12‐well plate using progenitor outgrowth medium (POM), which consists of IMDM supplemented with 10% FBS, 1% penicillin‐streptomycin (GIBCO), 1 µM β‐estradiol (Sigma), and 25 ng/mL recombinant mouse Flt‐3 ligand for generation of Hoxb8‐FL cells. Cells were infected with MSCV‐ERHBD‐Hoxb8 vector by spin inoculation. The MSCV‐ERHBD‐Hoxb8 plasmid was a generous gift from Hans Haecker (St. Jude Faculty, Department of Infectious Diseases, St. Jude Children's Research Hospital, Memphis, USA). Once established, Hoxb8‐FL cell lines were transduced with pMIG‐JAK2^V617F^ or pMIG‐JAK2^WT^ retroviral vectors and sorted on GFP+ expression to establish pure cultures. HoxB8‐FL cells were maintained in IMDM (GIBCO) supplemented with 10% FBS, 1% P/S, 40 ng/mL recombinant mouse Flt‐3 ligand (Peprotech), and 1 µM β‐estradiol (Sigma).

### In vitro coculture assays of HoxB8‐Flt3 cells and primary MSCs

Primary stromal cells isolated from C57BL/6J mice were cultured in αMEM with 20% MSC fetal calf serum (GIBCO), 1% penicillin‐streptomycin, 5 ng/mL endothelial growth factor (Peprotech), and 1 ng/mL fibroblast growth factor (Peprotech). HoxB8 cells were then washed and cocultured for 72 h with Gli1+ stromal cells, seeded at a density of 50,000 cells/well in alphaMEM containing 10% MSC‐qualified FBS. For the analysis of stromal cells, hematopoietic cells were harvested, wells were washed four times with PBS, and stromal cells were recovered by trypsinization.

### In vitro treatment of SET‐2 cells with tasquinimod

Human SET‐2 cells were cultured in RPMI media (GIBCO) with 20% FCS and 1% penicillin‐streptomycin. SET‐2 cells were treated with either 50 µM tasquinimod or its DMSO control, every 24 h for 48 h. Cells were then harvested, washed, and resuspended in Trizol for RNA extraction.

### RNA extraction and real‐time qRT‐PCR analysis

RNA from pelleted MSCs was extracted using Trizol solution (ThermoFisher) according to the manufacturer's instructions, and 1 μg of total RNA was reverse transcribed with Superscript IV (Invitrogen). Quantitative polymerase chain reactions were performed with SYBRGreen PCR master mix (ThermoFisher) on an Applied Biosystems 7500 Real‐Time PCR System. Glyceraldehyde‐3‐phosphate dehydrogenase (Gapdh) was used as a housekeeping gene. Data were analyzed using the 2−ΔΔct method.

### Statistical analysis

Statistical analysis was performed using GraphPad Prism v9 software (GraphPad Software Inc., San Diego, CA). Comparison between two groups was performed using an unpaired *t*‐test or Mann–Whitney test as described in the figure legends. For multiple group comparison, an analysis of variance (ANOVA) with post hoc Tukey correction or a Kruskal–Wallis test was applied. For multiple group comparisons over time, a two‐way ANOVA was performed with a mixed effect analysis, followed by a Tukey's multiple comparisons test. Data are shown as data ± SEM, and a P‐value of less than 0.05 was considered statistically significant.

## AUTHOR CONTRIBUTIONS

**Hélène F. E. Gleitz**: Conceptualization; investigation; writing—original draft; validation; visualization; writing—review and editing; formal analysis; supervision; data curation; methodology; funding acquisition. **Stijn N. R. Fuchs**: Investigation; writing—review and editing; methodology; validation. **Inge A. M. Snoeren**: Writing—review and editing; investigation; methodology. **Charlotte Boys**: Writing—review and editing; investigation; software; formal analysis; methodology; data curation; visualization. **James Nagai**: Investigation; software; writing—review and editing; methodology; data curation; visualization. **Hector Tejeda‐Mora**: Investigation; software; visualization; writing—review and editing; methodology; data curation. **Vanessa Klöker**: Investigation; software. **Jessica E. Pritchard**: Investigation; validation. **Iris J. Bakker**: Investigation; validation. **Marta Gargallo Garasa**: Investigation; validation. **Eric Bindels**: Writing—review and editing; resources; methodology. **Julio Saez‐Rodriguez**: Supervision; writing—review and editing; methodology. **Thomas Vogl**: Writing—review and editing; resources. **Rafael Kramann**: Supervision; writing—review and editing; conceptualization; resources. **Aurélien Dugourd**: Supervision; software; formal analysis; conceptualization; data curation; writing—review and editing; visualization. **Ivan G. Costa**: Formal analysis; software; supervision; data curation; writing—review and editing; methodology. **Rebekka K. Schneider**: Conceptualization; funding acquisition; writing—original draft; writing—review and editing; project administration; resources; supervision; visualization; data curation.

## CONFLICT OF INTEREST STATEMENT

R.K., I.C., and R.K.S. are members of the E:MED Consortia Fibromap and the consortium CureFib funded by the German Ministry of Education and Science (BMBF). Tasquinimod was generously provided by Active Biotech AB, Sweden. R.K.S. is a consultant for Active Biotech AB, Sweden, and received a research grant from them. R.K.S. and R.K. are cofounders and shareholders of Sequantrix GmbH. For the remaining authors, no relevant conflicts of interest were declared.

## FUNDING

H.F.E.G. is supported by a Gilead Research Scholar Award in Oncology/Hematology, a ZonMW VENI grant, and an Erasmus Medical Center Fellowship. R.K.S. is supported by the Oncode Institute and is supported by ERC grants (Rewind‐MF ERC‐CoG 101124542; deFIBER ERC‐StG 757339 and PoC DeAlarmin) and a ZonMW VIDI grant. This work was in part supported by grants of the Deutsche Forschungsgemeinschaft (DFG) (German Research Foundation) to H.F.E.G. (*417911533*), R.K. (KR 4073/9‐1), R.K.S. (504777725, 417911533, and 514007497), and I.C. (*417911533*). The project also received funding from the program “Netzwerke 2021,” an initiative of the Ministry of Culture and Science of the State of Northrhine Westphalia (CANTAR network). Open access funding enabled and organized by Projekt DEAL.

## Supporting information

Supporting Information.

Supplementary Information

Supplementary Information

Supplementary Information

Supplementary Information

Supplementary Information

## Data Availability

The data that support the findings of this study are openly available in Zenodo at https://zenodo.org/10.5281/zenodo.13740240.
