# Supplementary material for: Inhibiting the alarmin‐driven hematopoiesis‐stromal cell crosstalk in primary myelofibrosis ameliorates bone marrow fibrosis
Source: Hemasphere. 2025 Aug 14;9(8):e70179. doi: 10.1002/hem3.70179 (PMC12351185; doi:10.1002/hem3.70179)
Supplement: Supplementary file 1 — Supporting Information. [file HEM3-9-e70179-s001.docx]

**Supplementary figures and figure legends**

**
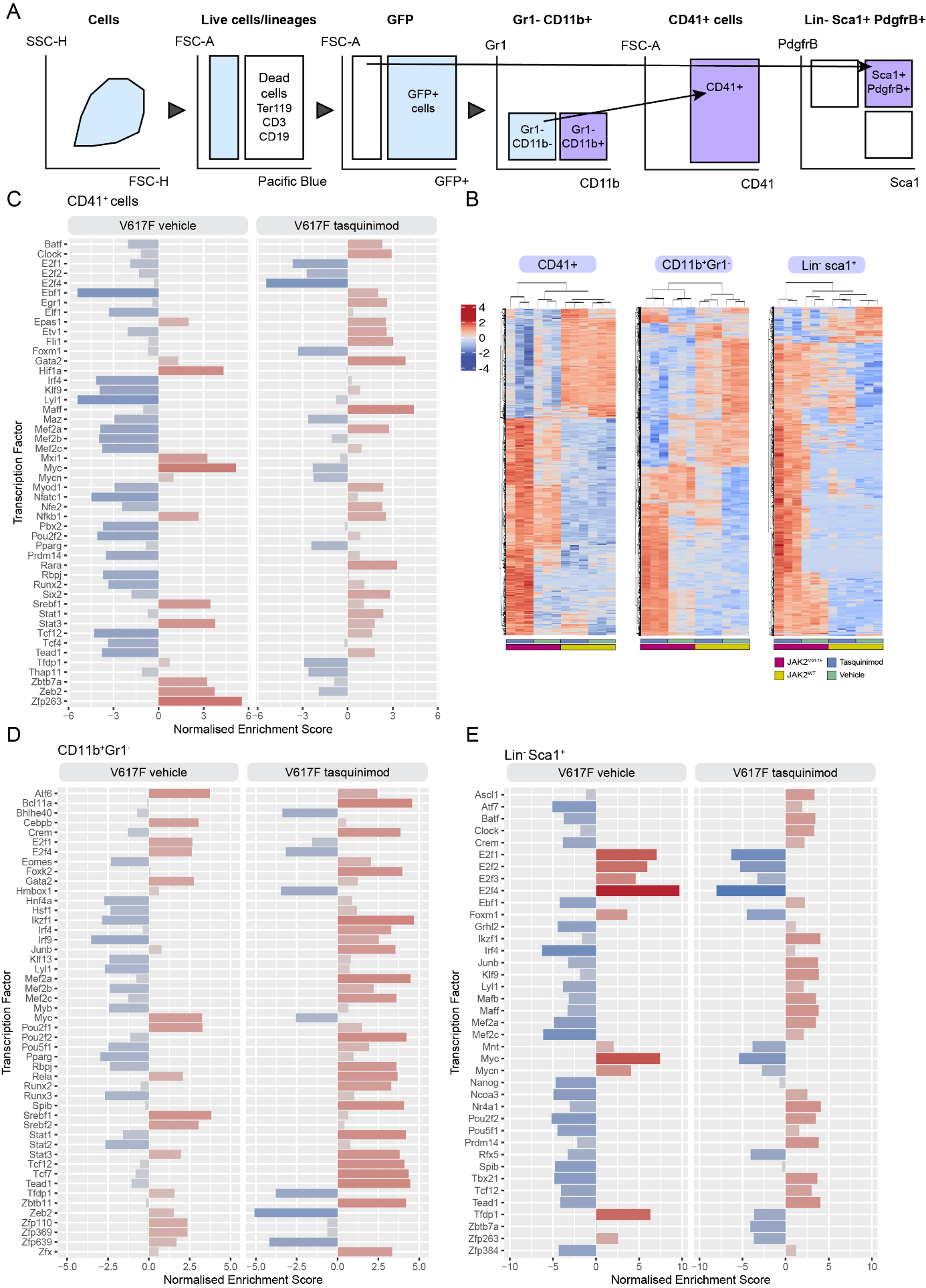
**

**Supplementary figure 1.** (A) Schematic representation of sorting strategy for monocytes, CD41+ cells and lin- Sca1+PdgfrB+ stromal cells. (B) Heatmap representation of significantly dysregulated genes of megakaryocytes, monocytes and stromal cells in JAK2^WT^ or JAK2^V617F^, untreated or treated with tasquinimod. DOROThEA analysis of transcription factors in (C) CD41+ cells, (D) CD11b+ Gr1- monocytes and (E) Lin-Sca1+ cells sorted from mice harboring the JAK2WT or JAK2V617F mutation, untreated or treated with tasquinimod. All comparisons are JAK2V617F vehicle vs. JAK2WT vehicle (labelled as “V617F vehicle”) and JAK2V617F Tasquinimod vs. JAK2V617F vehicle (labelled as “V617F Tasquinimod, n=3 mice/group/cell population).

**
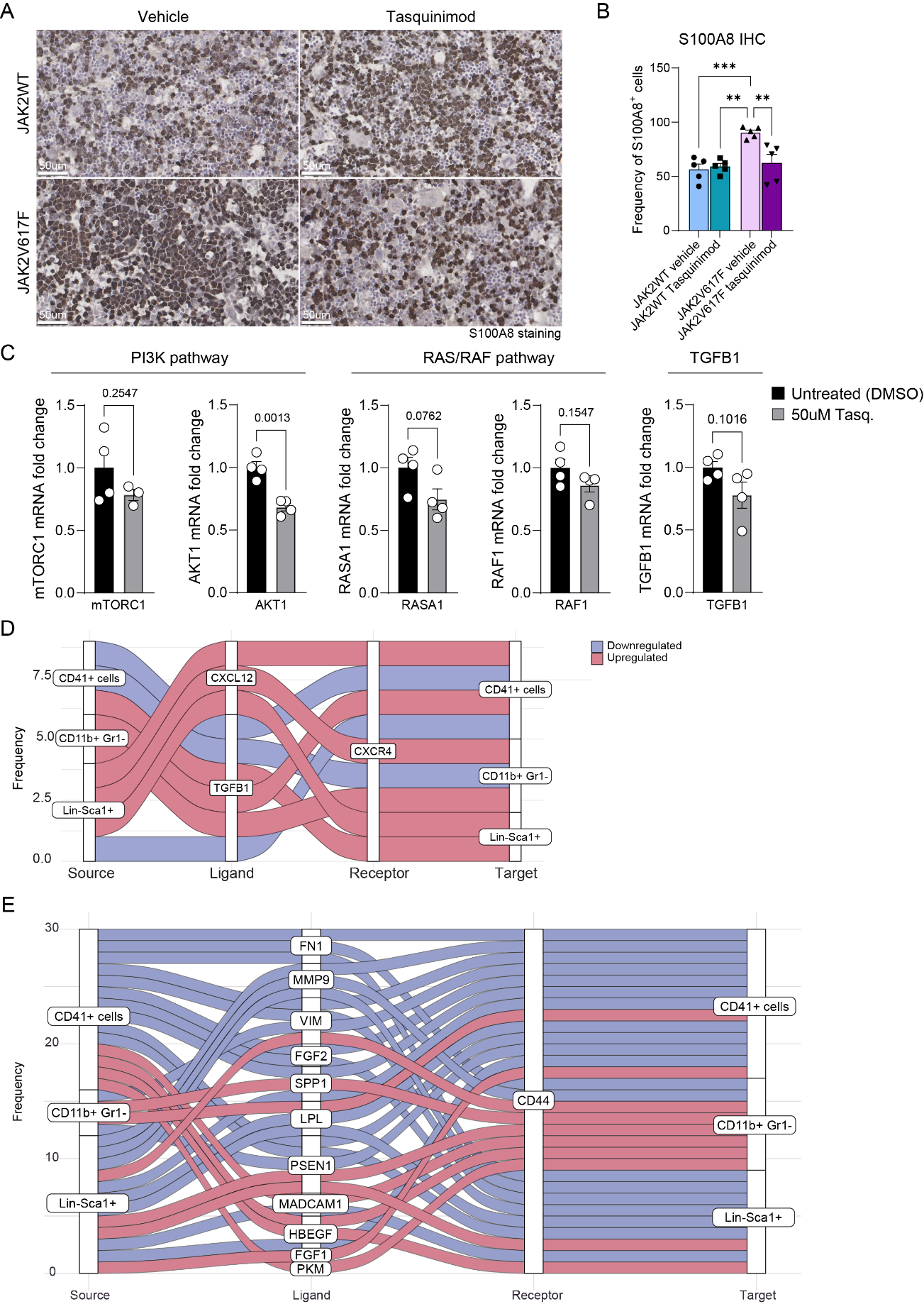
**

**Supplementary figure 2**. (A) Quantification of S100A8+ hematopoietic cells in Tasquinimod- or control-treated JAK2^V617F^ mice. Scale bar: 50 µm. (B) Representative images of S100A8 staining in 4um femoral section of Tasquinimod- or control-treated JAK2^V617F^ mice (or JAk2WT control mice). (C) qPCR analysis of mTORC1, AKT1, RASA1, RAF1 and TGFB1 in human SET-2 cells treated every 24 hours for 48 hours with 50uM tasquinimod or DMSO control. n=4/group. (D) Deregulated interactions mediated by CXCL12 and TGFB1 between CD41^+^, CD11b^+^Gr1^-^ and Lin^-^Sca1^+^ cells. Interactions ordered based on difference in mean LR expression between JAK2^V617F^ Tasquinimod and JAK2^V617F^ vehicle. (E) Top 30 deregulated interactions mediated by the CD44 receptor between CD41^+^, CD11b^+^Gr1^-^ and Lin^-^Sca1^+^ cells. Interactions ordered based on difference in mean LR expression between JAK2^V617F^ Tasquinimod and JAK2^V617F^ vehicle.


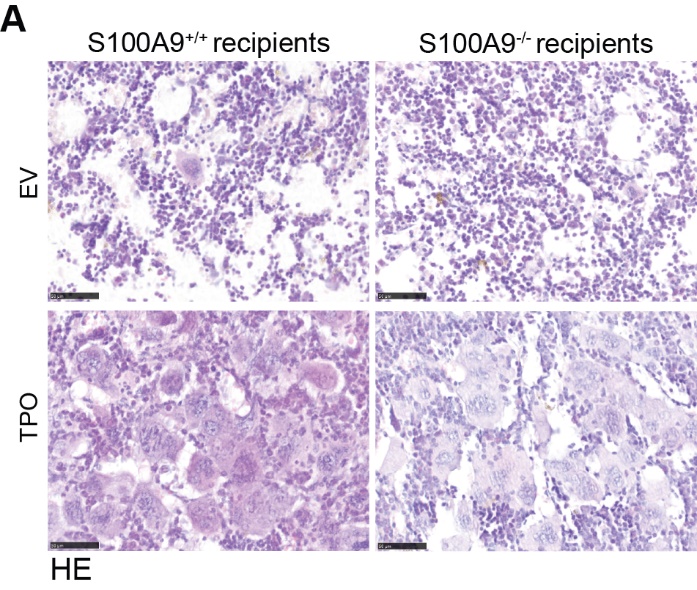


**Supplementary figure 3.** (A) Representative images of HE staining in femurs of WT or S100A9^-/-^ TPO-driven MPN mice. Scale bar: 50µm.


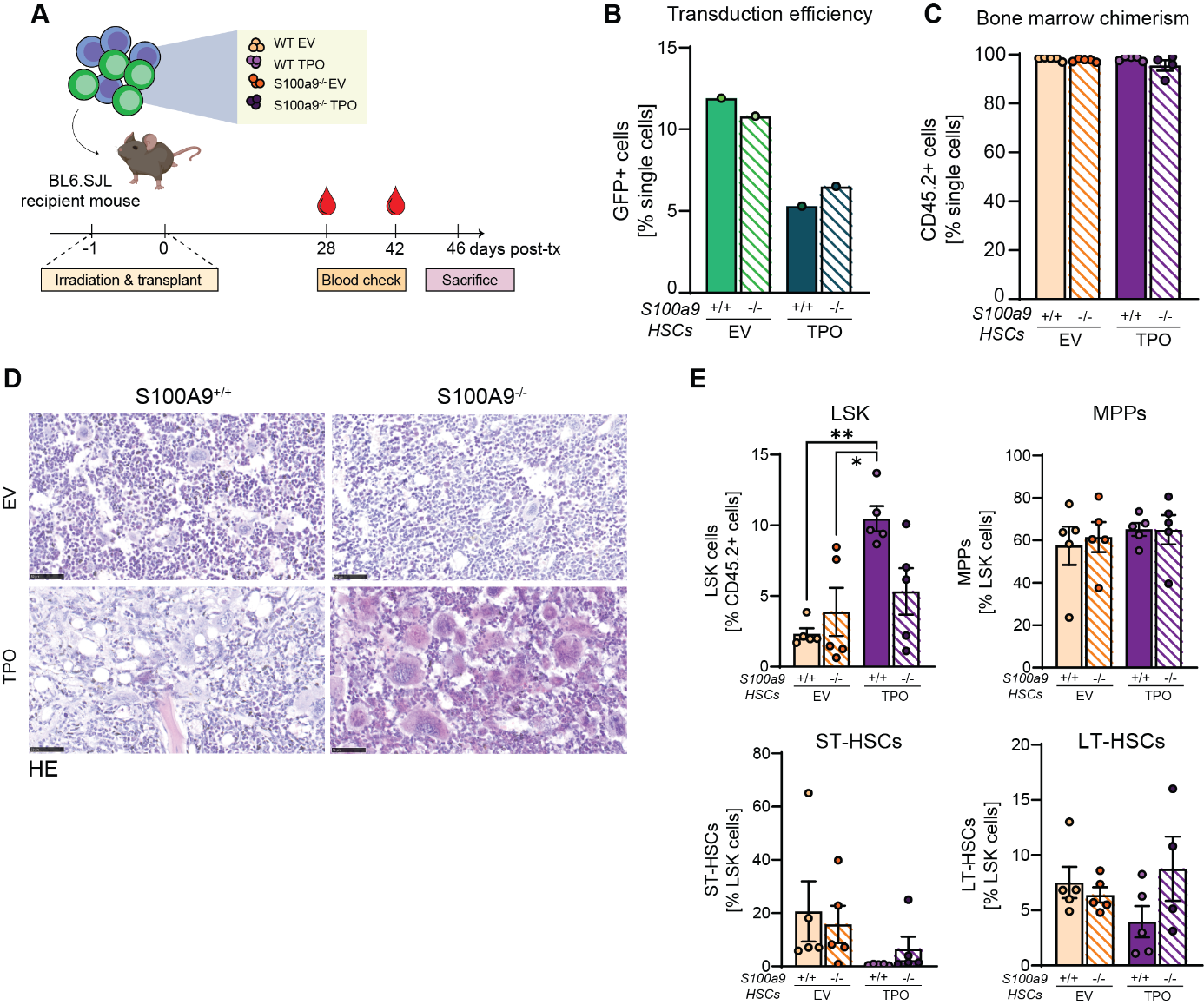


**Supplementary figure 4.** (A) Schematic representation of transplantation of WT or S100a9^−/−^ ckit^+^ cells in a model of TPO-induced fibrosis (n = 5, WT TPO; n = 4, S100a9^−/−^ TPO). (B) Transduction efficiency of TPO-GFP or EV-GFP in WT or S100a9^-/-^ ckit+ cells transplanted into recipient animals. (C) Bone marrow chimerism (donor (CD45.2+) vs. recipient (CD45.1+) cells) in recipient animals. (D) Representative images of HE staining in femurs of WT or S100a9^−/−^ TPO or EV mice. (E) Frequency of LSK, MPPs, ST-HSCs and LT-HSCs in WT or S100a9^−/−^ TPO or EV mice.


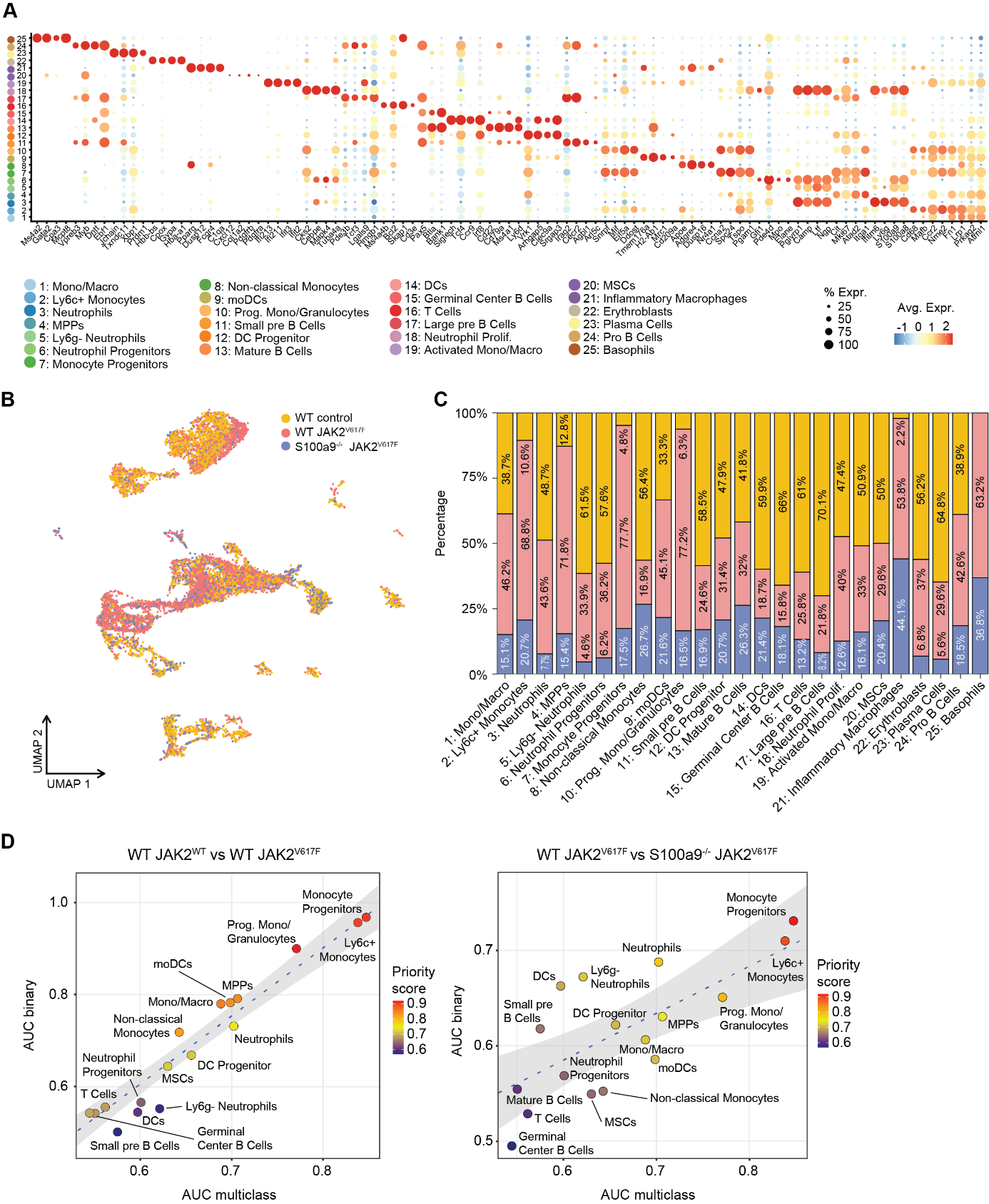


**Supplementary figure 5. (**A) Dotplot showing the expression of some of the marker genes used to validate cell type annotation in the single-cell RNA-seq dataset. Dot size indicates the proportion of cells within a cell type expressing the gene, while color intensity reflects the average expression level among expressing cells. Data was not shown if the percentage of cells expressing the gene was lower than 0.01. (B) UMAP visualization showing single cells used in the analysis, grouped by experimental conditions. (C) Bar plot showing the relative proportions of identified cell types across conditions. Cell proportions are calculated as percentages of total cells per cell type. MPPs: multipotent progenitor cells; moDCs: monocyte-derived dendritic cells; DC: dendritic cells; MSCs: mesenchymal stromal cells. (D) Scatter plot showing cell prioritization. Each point represents a cell type. The x-axis shows the area under the receiver operating characteristic curve (AUC) for the multiclass comparison, while the y-axis shows the AUC for the pair comparison. Higher AUC values indicate greater separability and thus higher prioritization.
