# Supplementary figures and images for: Inhibiting the alarmin‐driven hematopoiesis‐stromal cell crosstalk in primary myelofibrosis ameliorates bone marrow fibrosis
Source: Hemasphere. 2025 Aug 14;9(8):e70179. doi: 10.1002/hem3.70179 (PMC12351185; doi:10.1002/hem3.70179)

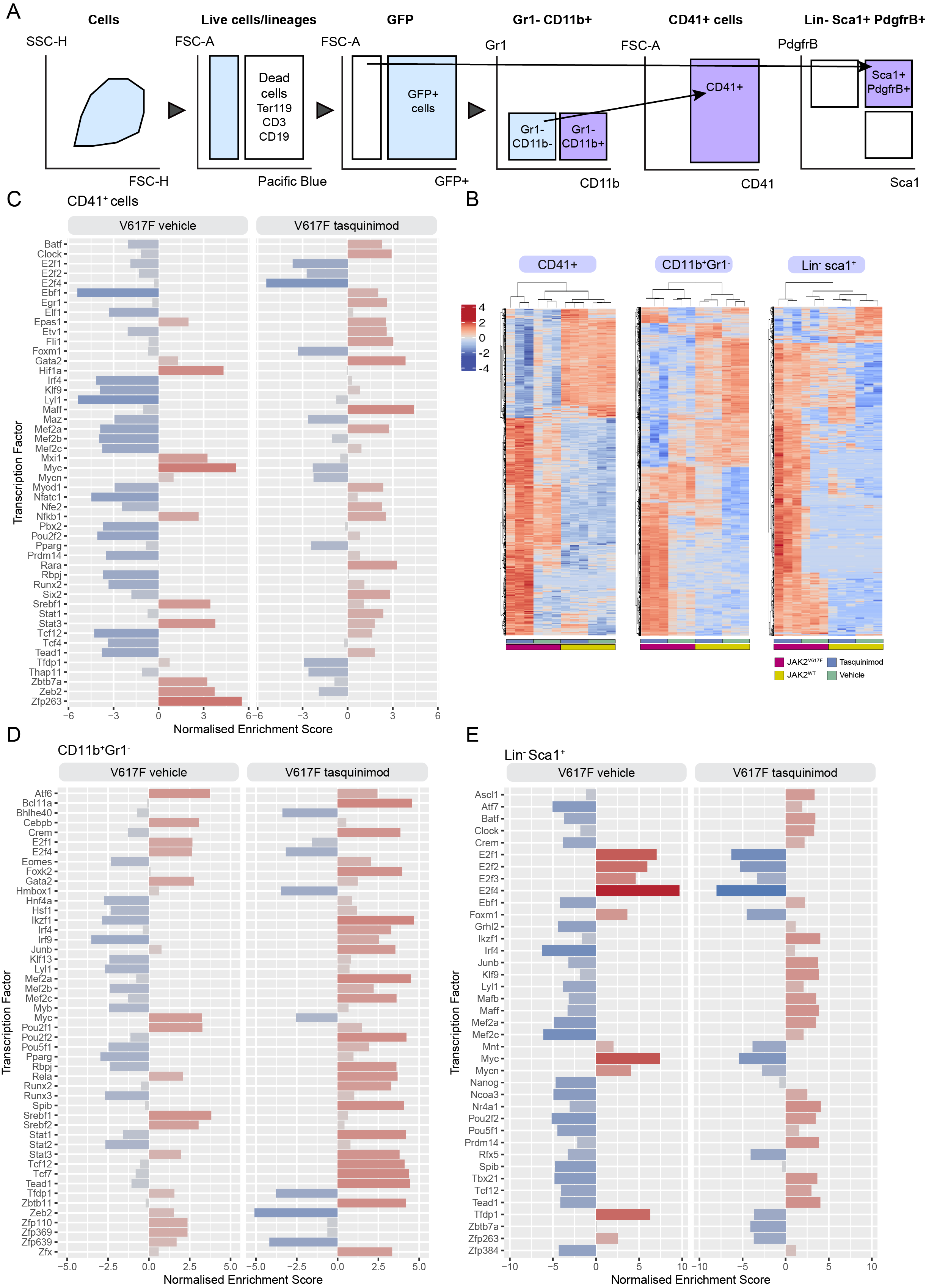

Supplement: Supplementary file 2 — Supplementary Information [file HEM3-9-e70179-s006.png]

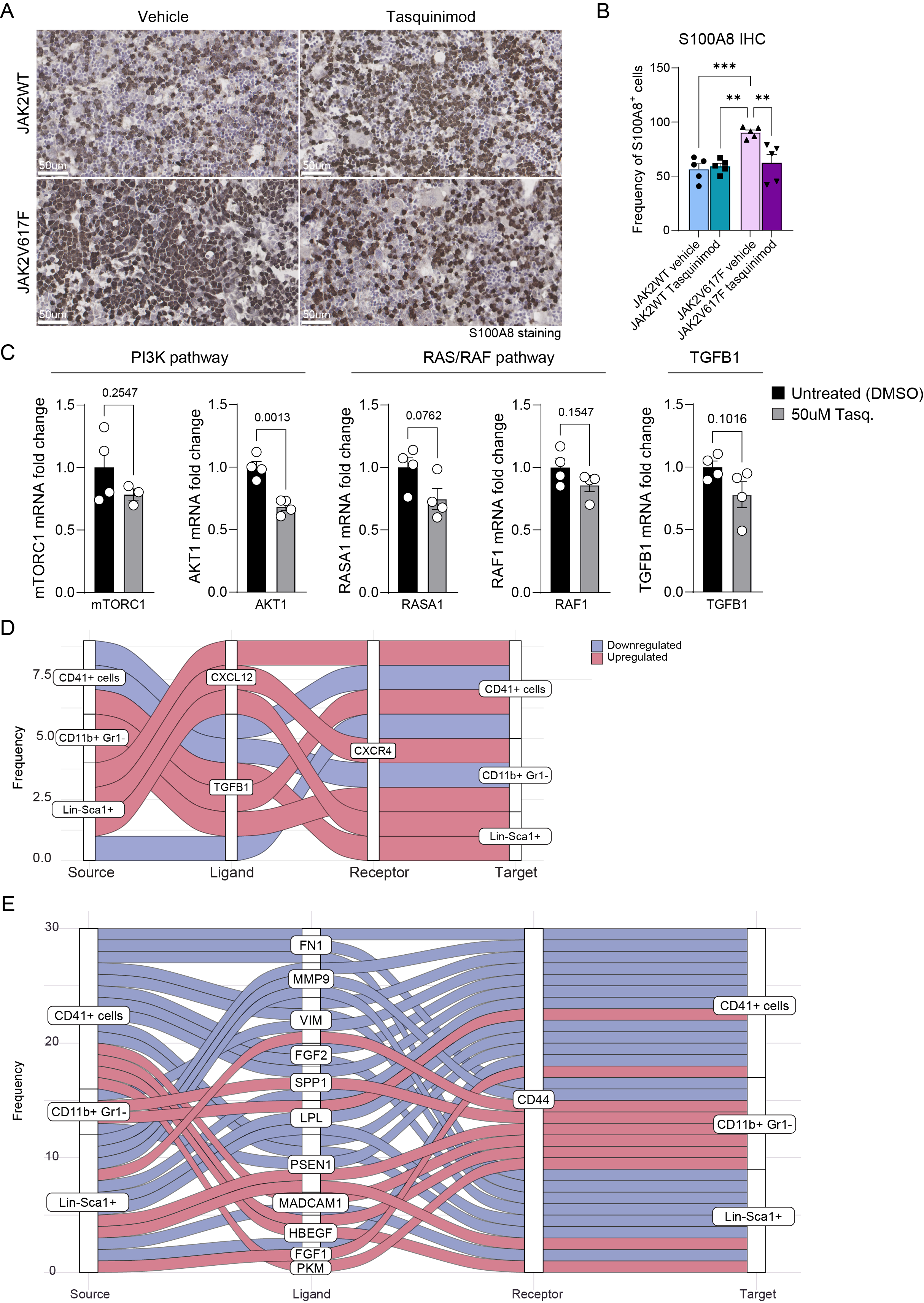

Supplement: Supplementary file 3 — Supplementary Information [file HEM3-9-e70179-s003.png]

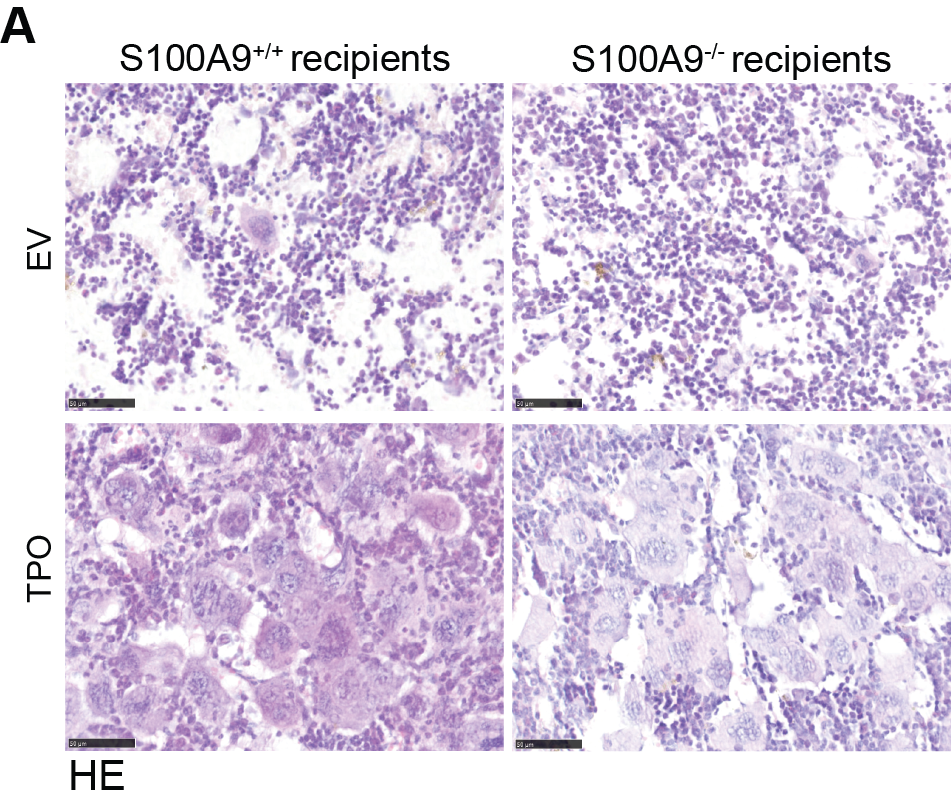

Supplement: Supplementary file 4 — Supplementary Information [file HEM3-9-e70179-s005.png]

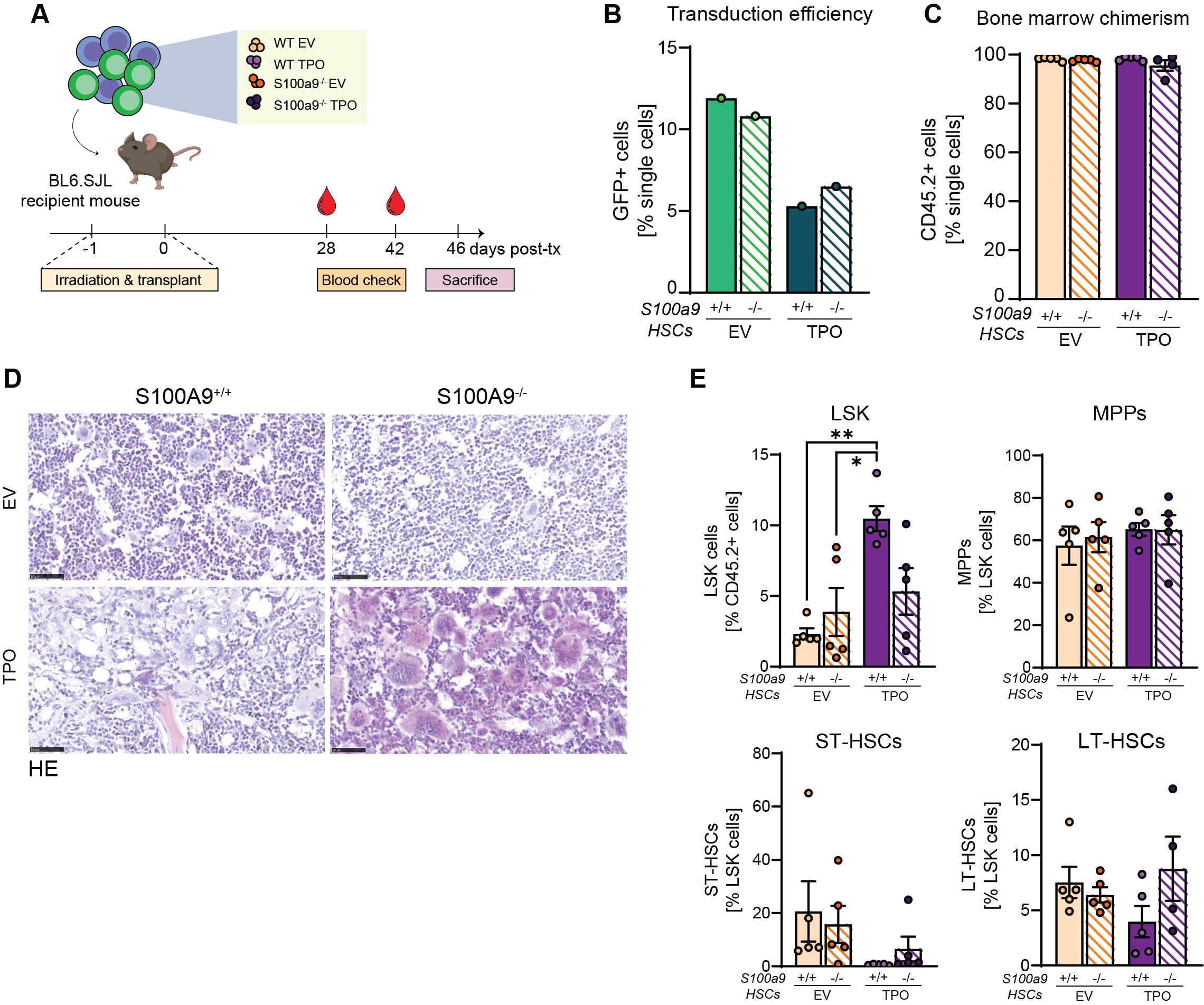

Supplement: Supplementary file 5 — Supplementary Information [file HEM3-9-e70179-s004.png]

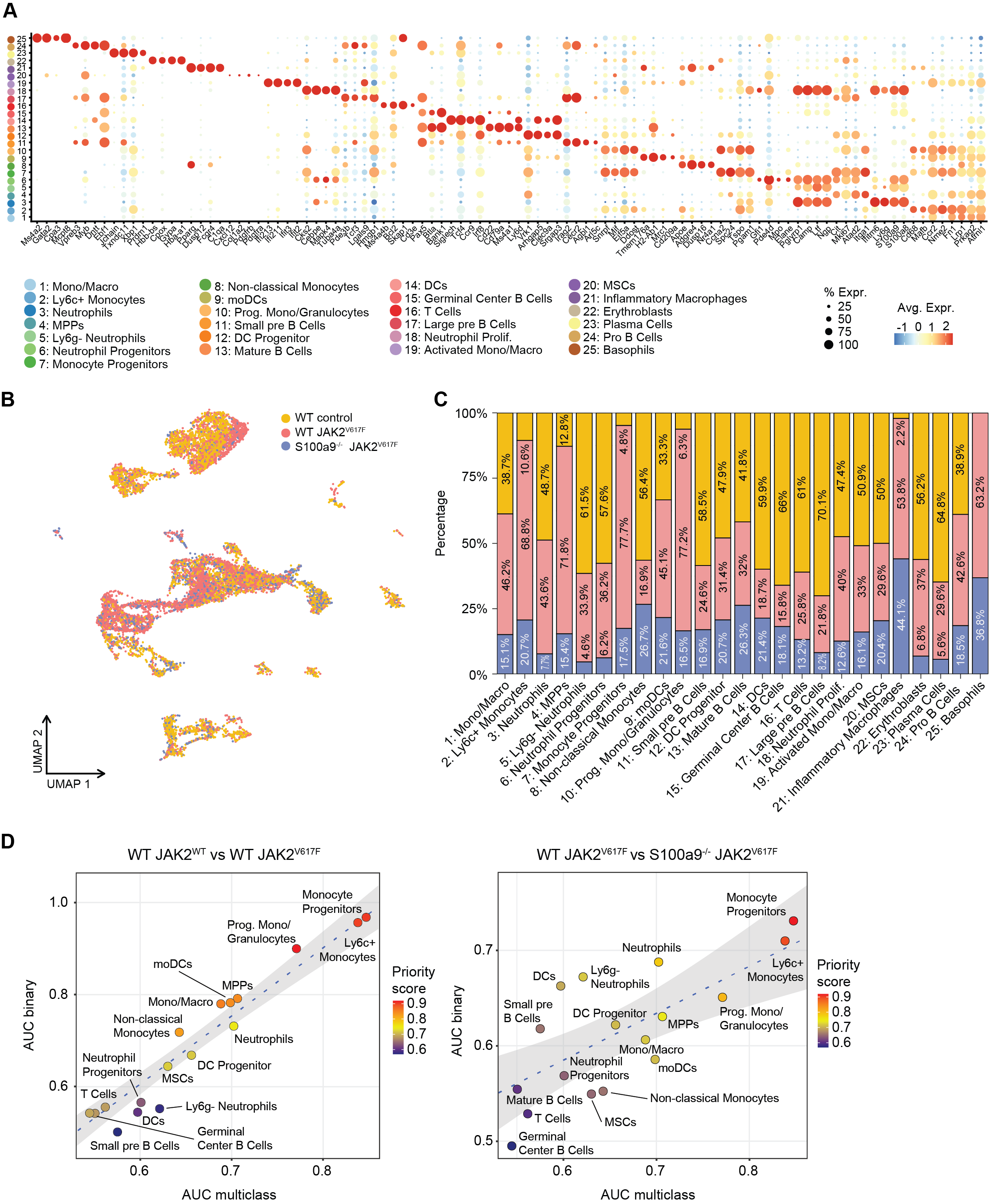

Supplement: Supplementary file 6 — Supplementary Information [file HEM3-9-e70179-s002.png]
